# Structural characteristics, cation distribution, and elastic properties of Cr^3+^ substituted stoichiometric and non-stoichiometric cobalt ferrites†

**DOI:** 10.1039/d1ra09090a

**Published:** 2022-03-17

**Authors:** M. A. Islam, A. K. M. Akther Hossain, M. Z. Ahsan, M. A. A. Bally, M. Samir Ullah, S. M. Hoque, F. A. Khan

**Affiliations:** Department of Physics Bangladesh University of Engineering and Technology Dhaka 1000 Bangladesh maislam.buet.phy@gmail.com; Departments of Chemistry and Physics, Gono Bishwabidyalay Savar Dhaka-1344 Bangladesh; Materials Science Divisions, Atomic Energy Center Dhaka-1000 Bangladesh

## Abstract

Structural, elastic and cation distribution properties have been investigated on stoichiometric and non-stoichiometric cobalt ferrites. Crystal structure, formation of spinel type ferrite, chemical bonding, cation distribution, and thermal properties of two series of Cr^3+^ substituted stoichiometric and non-stoichiometric various cobalt ferrites with general formula Co_1−*x*_Cr_*x*_Fe_2_O_4_ (S1), and Co_1+*x*_Cr_*x*_Fe_2−*x*_O_4_ (S2) were reported. Samples are synthesized by the solid-state reaction technique *via* planetary ball milling. X-ray diffraction (XRD) analysis confirms the formation of a single phase cubic spinel structure with the space group *Fd*3̄*m*. Rietveld refinement results show that Cr occupies both the tetrahedral (A-site) and octahedral sites (B-site). The experimental lattice parameters show increasing trends for both the series with increase of Cr content. The cation–anion vacancies, chemical bonding, and the displacement of oxygen have been evaluated to understand the effect of Cr substitution and how the non-stoichiometry affects the physical and chemical properties of the material. The crystallite size is found to be the decreasing value with an increase of Cr concentration for both series of samples. Specific vibrational modes from the FTIR spectra suggest a gradual change of inversion of the ferrite lattice with the increase of Cr concentration which is also evident from Rietveld refinement data. The elastic properties analysis reveals that the synthesized samples for both series are ductile in nature. The non-stoichiometric structure with excess Co^2+^ may pave a new way to realize the lowering of Curie temperature of ferrite that is expected to improve the magnetocaloric properties.

## Abstract 2

Structural, elastic and cation distribution properties have been investigated on stoichiometric and non-stoichiometric cobalt ferrites.

## Graphical abstract

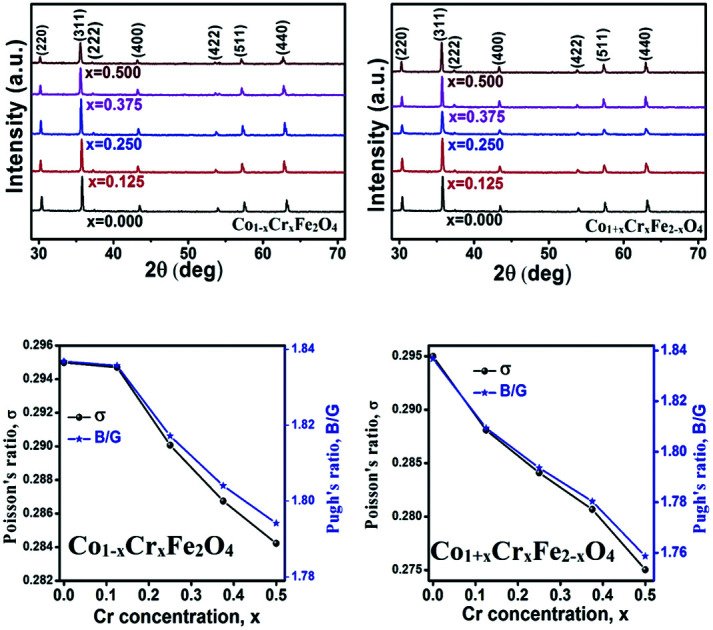

## 1. Introduction

Ferrites are ferrimagnetic materials usually used in high density recording media and satellite communication as well as microwave devices in electronic industries due to their ease of fabrication, low cost, moderate saturation magnetization, mechanical strength, and chemical stability.^1^ In the last decade, the synthesis of spinel ferrites emerged extensively due to their diversified use in technological applications.^2,3^ In particular, cobalt ferrite (COF) has got enormous importance both in technological as well as biomedical applications.^3–13^ Intensive studies on COF have been carried out with varying doping and/or substituting the divalent or trivalent cations and synthesizing them by different methods.^3,14,15^ In order to confirm the crystallinity and the phase of the synthesized materials, Rietveld refinement has been significantly used on the X-ray diffraction (XRD) data of the samples.^1,9–11,16^ Amri *et al.*^1^ have calculated the theoretical lattice parameters along with hopping lengths, oxygen positional parameters, cation–cation and cation–anion bond lengths of Ni–Zn–Al ferrite. Kumar *et al.*^14^ have reported on the estimation of the lattice parameters by using Nelson–Riley (N–R) function, intrinsic strain and crystallite size by using Williamson–Hall (W–H) method. It has also been studied that the crystallite size and intrinsic strain can be determined from the XRD peak broadening analysis by using several ways such as W–H method, size strain plot (SSP), Scherer method, and the modified Scherer method (MS).^14,17,18^ However, it is reported that SSP and MS methods are found more significant compared to that of W–H and Scherer methods, because both SSP and MS can be observed at a lower angle where the accuracy is found to be very high.^18^ On the other hand, the formation of ferrite and identification of the chemical bonds can be analyzed from the Fourier transform infrared (FTIR) spectra.^1,19–21^ Several techniques for the measurement mechanical properties have been used to calculate the elastic and mechanical properties. FTIR is one of the suitable techniques found to calculate the elastic and thermal properties. The elastic moduli, Debye temperature, and minimum thermal conductivity can be calculated by using stiffness, lattice parameters, and X-ray density which have been reported in various literatures^1,19,20,22^ Chandekar, *et al.*^22^ reported the corrected zero porosity elastic moduli estimated from the calculated elastic moduli and porosity.

The synthesis and characterization of ferrites have attracted increasing attention in recent years. Many efforts have been attempted to expand potential applications by improving their properties. Research on inorganic compounds with a non-stoichiometric ratio is a great challenge. Andersen *et al.*^23^ reported the local and long-range magnetic structure of non-stoichiometric nanocrystalline spinel type iron oxide. They have found the vacancy ordering in the structure which reveals the shorter coherence length on the non-stoichiometric spinel type iron oxide. In addition various literature on non-stoichiometric compounds have been found,^24–28^ where remarkable effects of non-stoichiometry on structural, and magnetic properties have been presented.

This study focuses on how Cr^3+^ affect the structure of stoichiometric COF with the formula Co_1−*x*_Cr_*x*_Fe_2_O_4_ (S1) and non-stoichiometric COF having the formula Co_1+*x*_Cr_*x*_Fe_2−*x*_O_4_ (S2), where *x* = 0.125, 0.25, 0.375, and 0.50. The cation distribution has been calculated using site occupancy data extracted from the Rietveld refined XRD data. Besides, the theoretical and experimental lattice parameters, oxygen positional parameter, tolerance factor, ionic packing coefficient, vacancy parameter, bond lengths, bond angles, density, and porosity of the above-mentioned samples of both the series have been determined. Crystallite size estimation by various methods such as W–H, SSP, Scherer, MS, and Rietveld methods has also been explained in this study. FTIR spectra have been illustrated to confirm the ferrite formation for the samples of both the investigated series. In addition, elastic and thermal properties have also been explained from the FTIR and structural data. A correlation between the elastic properties and structural properties has been established for exploiting their advanced applications. The main focus of this work is to find the structural defects of COF with Cr^3+^ substitution at the Co^2+^ site. In addition, effects of non-stoichiometry on the structure of COF are also explored.

## 2. Experimental procedure

### 2.1 Synthesis technique

The selected two series of stoichiometric and non-stoichiometric COF denoted as S1 and S2 have been synthesized by the standard solid-state reaction method. The required amount of Co_2_O_3_ (98.0%), Cr_2_O_3_ (99.9%), and Fe_2_O_3_ (96.0%) are weighed in an analytical balance and hand mixed in a mortar pestle for 2 h. The mixed powders are milled in a planetary ball mill (MSK-SFM-1) for 12 h. The milled powders are calcined at 800 °C for 6 h for a complete solid-state reaction through diffusion of particles. Then the powders of each composition are pressed into pellets by applying a hydraulic pressure of 16 000 psi. Finally, the pellets are sintered at 1200 °C for 6 h in a furnace and rcrushed in to fine powders.

### 2.2 Characterizations

The X-ray diffraction (XRD) data of the synthesized samples are taken using a Rigaku Smart Lab X-ray diffractometer with Cu-Kα radiation (*λ* = 1.5406 Å) with the scanning angle 2*θ* within the range of 10 to 80°. The Rietveld refinement was carried out using the Full-Prof software integrated in Match-3 software. Room temperature Fourier transform infrared (FTIR) spectroscopy (Spectrometer model-IR Prestige-21) in the region 350–4000 cm^−1^ was used to find out the functional groups and vibrational structure of the synthesized samples. The parameters related to structural, elastic, and thermal properties are calculated using the XRD data and FTIR spectra.

## 3. Results

### 3.1 XRD analysis

**Fig. 1:**
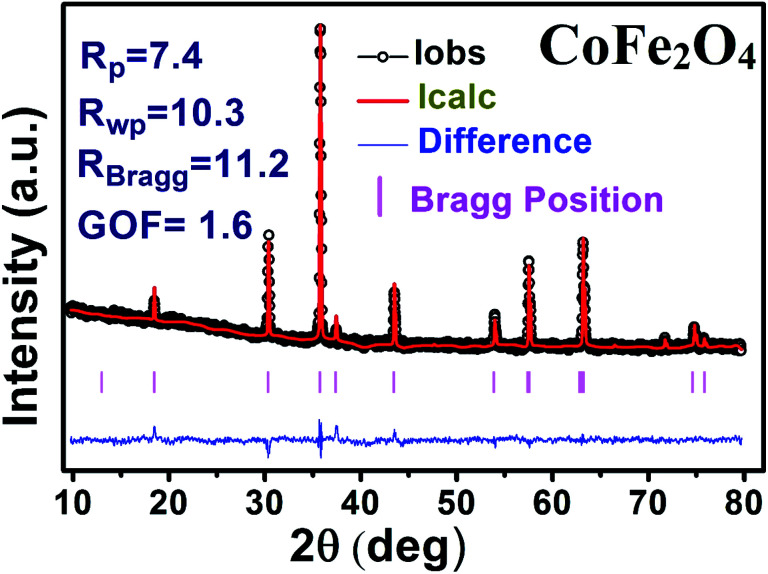
Rietveld analysis of XRD pattern of CoFe_2_O_4_.

**Fig. 2:**
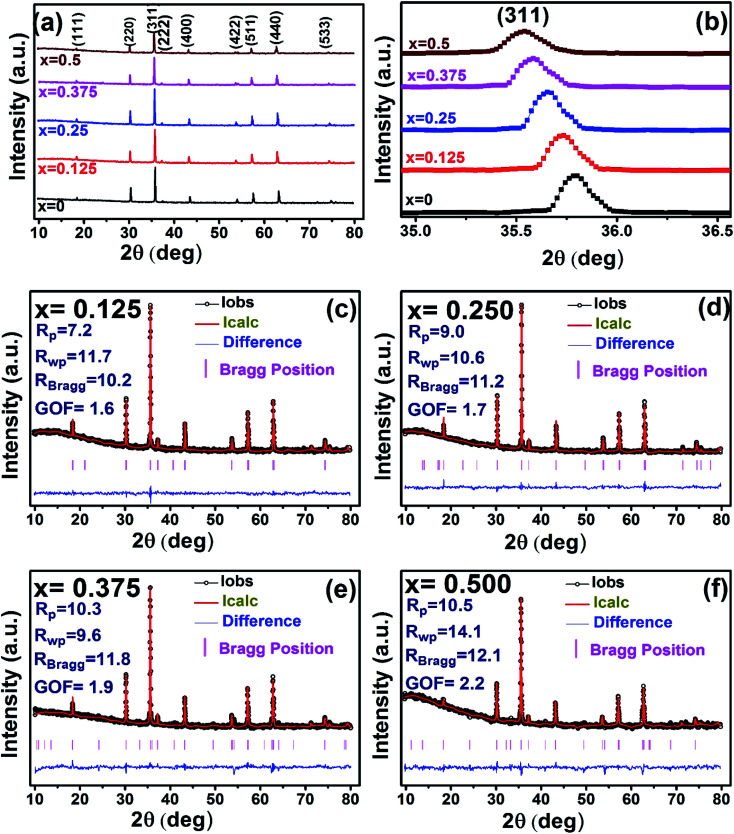
Rietveld analysis of XRD patterns for all samples of S1 (Co_1−*x*_Cr_*x*_Fe_2_O_4_) series.

**Fig. 3:**
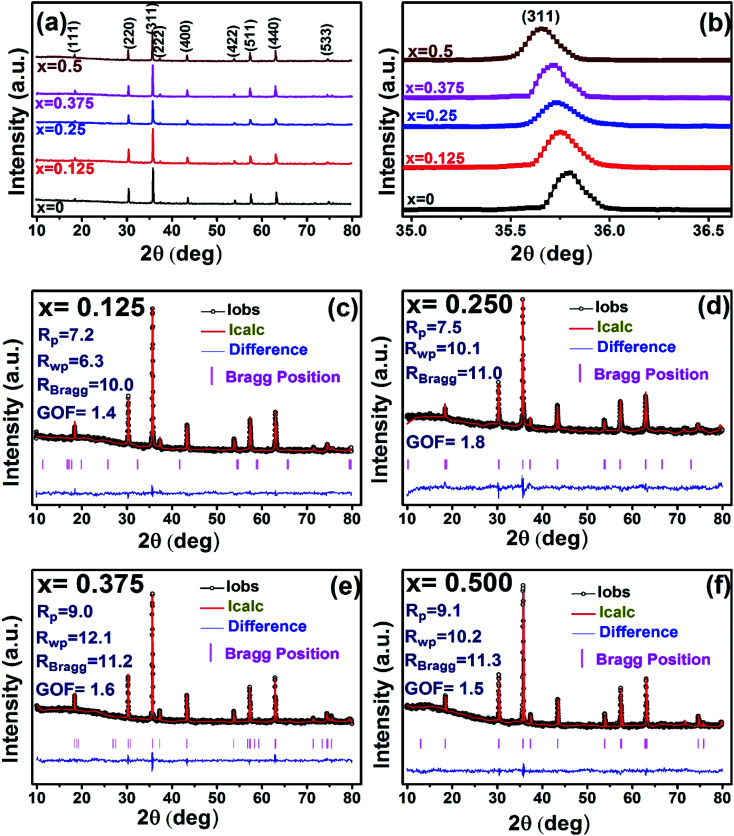
Rietveld analysis of XRD patterns for all samples of S2 (Co_1+*x*_Cr_*x*_Fe_2−*x*_O_4_). series.

The X-ray diffraction patterns along with Rietveld refined data have been shown in Fig. 1, 2, and 3 for the parent COF, other samples of the series S1, and S2, respectively. XRD pattern of the parent COF satisfy the Bragg's condition at 2*θ* values of 30.37°, 35.76°, 43.4°, 53.84°, 57.4°, and 62.92° with corresponding crystal planes are indexed with (220), (311), (400), (511), and (440), respectively. These sharp peaks, corresponding to the aforesaid (*hkl*) planes indicate a single phased inverse spinel structure with space group *Fd*3̄*m* of parent COF that has been confirmed by the crystallography open database (COD No. 5910063) as reported in the literature.^29^ No impurity phase has been observed in the pattern for the parent COF which signifies the phase purity of COF. The XRD patterns for the samples of the stoichiometric series S1 and non-stoichiometric series S2 shown in Fig. 2 and 3, reveal the formation of spinel structure (COD No. 910063). The peak positions of (311) plane for both the series S1 and S2 have been shifted towards the lower 2*θ* angle with an increase of Cr content as shown in Fig. 2(b) and 3(b), which imply an increase of the lattice parameters as a result of Cr substitution.

#### 3.1.1 Estimation of cation distribution

The cation distribution of the samples at the tetrahedral and octahedral sites of spinel structure has been determined by comparing the observed X-ray intensities from the specific planes of (220), (440), (400), and (422). The ratios of observed and calculated intensities *viz. I*_220_/*I*_400_, *I*_220_/*I*_440_, *I*_422_/*I*_440,_ and *I*_400_/*I*_440_ are considered to evaluate the cation distribution. From the calculation of structure factor, it is reported that (220) and (440) are sensitive to cation distribution at the tetrahedral site, while (400) and (422) are sensitive to cation distribution at the octahedral site.^30^ The general formulae for cation distribution at the tetrahedral site and octahedral site for samples of the series S1, and S2, are as follows:Possible cation distribution of S1 series is [Co_*α*_Cr_*β*_Fe_*γ*_]^tet^[Co_1−*x*−*α*_Cr_*x*−*β*_Fe_2−*γ*_]^oct^O_4_Possible cation distribution of S2 series is [Co_*α*_Cr_*β*_Fe_*γ*_]^tet^[Co_1+*x*−*α*_Cr_*x*−*β*_Fe_2−*x*−*γ*_]^oct^O_4_where *α*,*β*, and  *γ* are the cationic parameters for the tetrahedral site and *α* + *β* + *γ* = 1. For COF *x* = *β* = 0. The cation distribution has been calculated by the above formulae and tabulated in Table 1. The value of the inversion parameter as determined by calculating the ratio between Fe^3+^ at the tetrahedral site and Fe^3+^ at octahedral site Fe_tetra_/Fe_octa_ has also been tabulated in Table 1. Using the exact cation distribution the ionic radius for the tetrahedral site (*r*_tet_), and octahedral site (*r*_oct_), have been calculated by the following relations:^16^*r*_tet_ = *αr*_Co_ + *βr*_Cr_ + *γr*_Fe_ for both the series,*r*_oct_ = 0.5 × {(1 − *x* − α)*r*_Co_ + (*x* − *β*)*r*_Cr_ + (2 − *γ*)*r*_Fe_} for S1,*r*_oct_ = 0.5 × {(1 + *x* − *α*)*r*_Co_ + (*x* − *β*)*r*_Cr_ + (2 − *x* − *γ*)*r*_Fe_} for S2,where *r*_Co_, *r*_Cr_, and *r*_Fe_ are the ionic radii of Co^2+^, Cr^3+^, and Fe^3+^, respectively. The calculated ionic radii for the tetrahedral site and octahedral site are tabulated in Table 1.

**Table 1:** Possible cation distribution for all samples of series S1 (Co_1−*x*_Cr_*x*_Fe_2_O_4_) and S2 (Co_1+*x*_Cr_*x*_Fe_2−*x*_O_4_)

Series		S1				S2				
*x*		0.000	0.125	0.250	0.375	0.500	0.125	0.250	0.375	0.500
Tetrahedral site(±2%)	Co	0.132	0.100	0.090	0.070	0.050	0.179	0.121	0.042	0.012
	Cr	0	0.057	0.110	0.166	0.232	0.011	0.012	0.018	0.008
	Fe	0.868	0.843	0.800	0.764	0.718	0.810	0.867	0.940	0.980
Octahedral site (±2%)	Co	0.868	0.775	0.660	0.555	0.450	0.946	1.129	1.333	1.488
	Cr	0	0.068	0.140	0.209	0.268	0.114	0.238	0.357	0.492
	Fe	1.132	1.157	1.200	1.236	1.282	1.065	0.883	0.685	0.520
Calculated	*I*_400_/*I*_440_	0.77	0.76	0.81	0.81	0.83	0.83	0.85	0.80	0.80
	*I*_220_/*I*_440_	0.98	0.82	1.11	1.05	1.06	1.14	1.08	1.05	0.80
Observed	*I*_400_/*I*_440_	0.74	0.75	0.72	0.76	0.78	0.84	0.90	0.78	0.77
	*I*_220_/*I*_440_	1.03	1.03	1.10	1.02	1.01	1.25	1.24	1.00	1.04
Inversion parameter (*y*)	0.76	0.72	0.66	0.62	0.56	0.76	0.98	1.37	1.88	
Ionic radius (Å)	*r*_tet_	0.63	0.64	0.65	0.66	0.68	0.64	0.63	0.62	0.61
	*r*_oct_	0.69	0.68	0.68	0.67	0.66	0.73	0.78	0.83	0.87

**Fig. 4:**
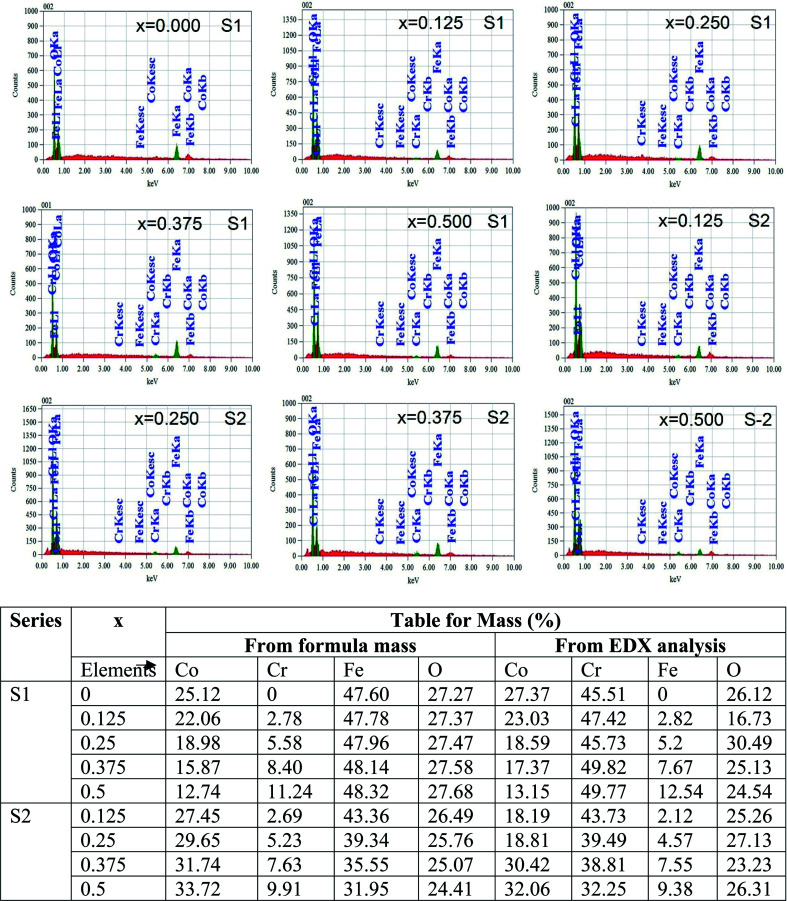
EDX spectra of all samples of S1 (Co_1−*x*_Cr_*x*_Fe_2_O_4_) and S2 (Co_1+*x*_Cr_*x*_Fe_2−*x*_O_4_). series with mapping of compositions.

Fig. 4 represents the Energy Dispersive (EDS) spectra for all samples. The elemental analysis are performed from these spectra. From the data of EDS spectra, it is confirmed that chemical composition of the synthesized samples are close to the respective nominal composition.

#### 3.1.2 Lattice parameter estimation

**Fig. 5:**
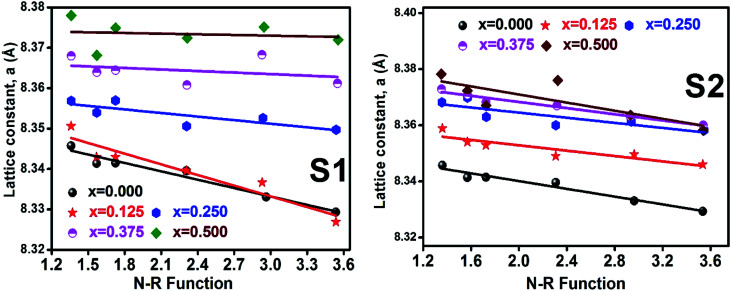
Nelson–Riley plot for all samples of series S1 (Co_1−*x*_Cr_*x*_Fe_2_O_4_) and S2 (Co_1+*x*_Cr_*x*_Fe_2−*x*_O_4_).

**Fig. 6:**
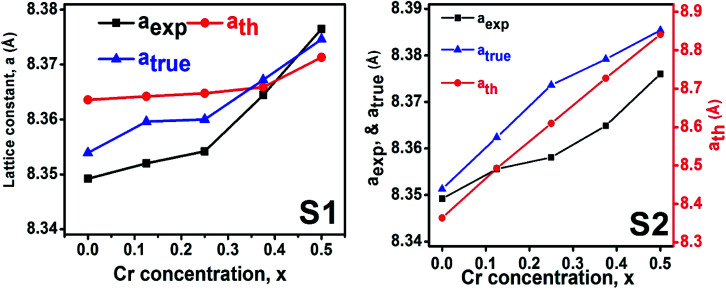
Lattice constants as function of Cr content for all samples of series S1 (Co_1−*x*_Cr_*x*_Fe_2_O_4_) and S2 (Co_1+*x*_Cr_*x*_Fe_2−*x*_O_4_).

To determine true value of lattice parameter (*a*_true_) from XRD data, Nelson–Riley (N–R) extrapolating function (eqn (A2)) has been used.^17^ The lattice parameter for each Bragg position has been calculated using eqn (A3). The N–R function *vs. a*_*hkl*_ graph has been plotted for each sample which is shown in Fig. 5 for both the series S1 and S2, respectively. From Fig. 5 the true value of lattice parameters for each sample have been determined by extrapolating *θ* = 0°. The extracted value of *a*_true_ for each sample have been illustrated in Fig. 6. The lattice parameters calculated from the XRD data by using Match-3 software are denoted by *a*_exp_, and these values for the both series of samples have been illustrated in Fig. 6. The lattice parameters calculated using the eqn (A1) are denoted by *a*_th_, and these values also have been illustrated in Fig. 6. It is observed that *a*_exp_ for the sample of both series follows the similar increasing trend of the *a*_th_ and *a*_true_ with the Cr content which implies that *a*_exp_ is reliable. This increase in lattice parameter for S1 series may be caused due to the larger ratio of Cr^2+^/Co^2+^ at tetrahedral site than the Cr^3+^/Co^2+^ ratio at octahedral site. The increasing trends of lattice parameter for S2 are due to the excess Co^2+^ with an ionic radius of 0.75 Å which is larger than that of Cr^3+^ (0.63 Å).^31^ Since unit cell volume is directly proportional to the lattice constant, hence it follows a similar trend of the lattice constant.

#### 3.1.3 Density and porosity estimation

**Fig. 7:**
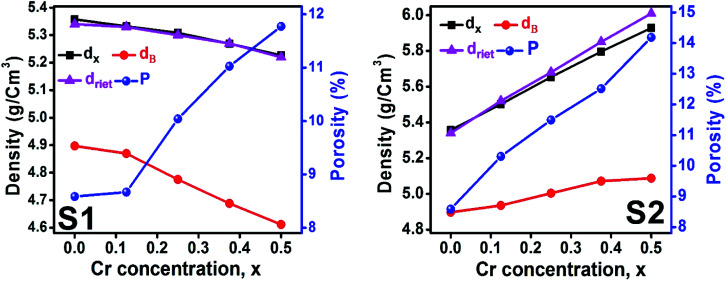
Density and porosity as function of Cr content for all samples of series S1 (Co_1−*x*_Cr_*x*_Fe_2_O_4_) and S2 (Co_1+*x*_Cr_*x*_Fe_2−*x*_O_4_).

The bulk density *d*_B_, crystal density *d*_x_, and porosity (*P*) for both series of samples have been calculated using eqn (A4), (A5), and (A6), respectively and have been illustrated as a function of Cr concentration in Fig. 7. The density extracted from the Rietveld refined data is denoted as *d*_reit_ is also presented in Fig. 7. These parameters have been listed in Table 2. It is seen that the *d*_x_, and *d*_B_ show a similar decreasing trend with the increase in Cr content which agrees with the Rietveld refined density *d*_reit_. The decreasing trend of the density is due to the molecular weight loss of the investigated samples. Furthermore, it is seen that the *d*_x_ and *d*_B_ follow the increasing trend with the Cr content that is also agrees with the *d*_reit_ for the samples of the series S2. This increasing nature of density may have been caused by the molecular weight gain due to the non-stoichiometry of samples of the series S2. The porosity is found to increase with the addition of Cr content for the samples of both series.

**Table 2:** Lattice parameter, density, and porosity for all samples of series S1 (Co_1−*x*_Cr_*x*_Fe_2_O_4_) and S2 (Co_1+*x*_Cr_*x*_Fe_2−*x*_O_4_)

Series	*x*	*a*_exp_ (Å)	*V*_exp_ (Å^3^)	*a*_true_ (Å)	*V*_true_ (Å^3^)	*a*_th_ (Å)	*V*_th_ (Å^3^)	*d*_x_ (g cm^−3^)	*d*_B_ (g cm^−3^)	*d*_reit_ (g cm^−3^)	*P* (%)
S1	0	8.3492	582.01	8.3539	582.45	8.3635	585	5.36	4.90	5.34	9
	0.125	8.3520	582.6	8.3596	579.51	8.3642	585	5.33	4.87	5.33	9
	0.250	8.3542	583.06	8.3600	578.15	8.3647	585	5.31	4.78	5.29	10
	0.375	8.3644	585.2	8.3672	577.47	8.3658	586	5.27	4.69	5.27	11
	0.500	8.3765	587.74	8.3746	576.55	8.3713	587	5.31	4.61	5.22	13
S2	0.125	8.3556	582.39	8.3705	586.48	8.4927	613	5.52	4.94	5.52	11
	0.250	8.3581	589.32	8.3896	590.50	8.6096	638	5.67	5.00	5.68	12
	0.375	8.3649	595.80	8.3958	591.81	8.7272	665	5.82	5.07	5.85	13
	0.500	8.376	604.68	8.4061	593.99	8.8414	691	5.95	5.09	6.01	15

#### 3.1.4 Oxygen positional parameter, interionic distance and bond angle estimation

When tetrahedral interstices are occupied by divalent ion then the expansion in tetrahedral site is relatively larger than octahedral site due to the difference of ionic radii of cations. This expansion can be explained by oxygen positional parameter, *u*, which gives quantitative measure of the displacement of oxygen ions. This displacement takes place whenever there is a difference in the radii of substituted and replaced ions. The value of *u* can be calculated from the *a*_th_ using the eqn (A7).

The calculated *u* values for both series are listed in Table 4. As seen in that table, *u* value is almost invariant with Cr content for both series found to be almost equal to that of the parent COF. In a cubic spinel structure the ideal value of *u* is equal to 3/8 = 0.375. The calculated value of *u* is slightly larger than that of ideal value, which may be due to the anion displacement from its ideal position. From the analysis of *u* and *δ* (= *u* − 0.375), it is confirmed that lattice is slightly distorted for all the samples of both series.^33^

The inter-ionic distance between magnetic ions is named as hopping length in tetrahedral site (*L*_A_) and octahedral site (*L*_B_) which gives information about the strength of spin interaction of ions. The hopping lengths have been calculated by using Stanley's equations (eqn (A8)).^1^ The calculated values of *L*_A_ and *L*_B_ have been tabulated in Table 3 for both the series. Hopping lengths decrease for S1 series, and increase for S2 series with the increasing Cr content.

**Table 3:** Values of hopping lengths (*L*_A,_*L*_B_), tetrahedral bond length (*d*_AL_), octahedral bond lengths (*d*_BL_), tetrahedral edge length (*d*_AE_), shared and unshared octahedral edge (*d*_BE_, *d*_BEU_), interatomic bond lengths and angles for all samples of series S1(Co_1−*x*_Cr_*x*_Fe_2_O_4_) and S2 (Co_1+*x*_Cr_*x*_Fe_2−*x*_O_4_)

Series		S1					S2			
*x*		0.000	0.125	0.250	0.375	0.500	0.125	0.250	0.375	0.500
*L*_A_ (Å)		3.623	3.622	3.617	3.616	3.614	3.667	3.717	3.768	3.809
*L*_B_ (Å)		2.958	2.957	2.953	2.952	2.951	2.994	3.035	3.077	3.11
*d*_AL_ (Å)		1.945	1.956	1.968	1.982	1.998	1.926	1.893	1.86	1.832
*d*_BL_ (Å)		2.011	2.006	2.000	1.996	1.993	2.023	2.042	2.063	2.083
*d*_AE_ (Å)		3.176	3.194	3.215	3.237	3.263	3.145	3.091	3.037	2.991
*d*_BE_ (Å)		2.727	2.711	2.692	2.677	2.659	2.762	2.818	2.877	2.931
*d*_BEU_ (Å)		2.956	2.958	2.959	2.964	2.969	2.957	2.956	2.958	2.961
Me–Me distances (Å)	*b*	2.9569	2.9572	2.9574	2.9577	2.9597	3.0026	3.0439	3.0855	3.1259
	*c*	3.4673	3.4676	3.4678	3.4683	3.4705	3.5209	3.5693	3.6181	3.6654
	*d*	3.6215	3.6218	3.6220	3.6225	3.6248	3.6774	3.7280	3.7789	3.8284
	*e*	5.4322	5.4327	5.4330	5.4337	5.4373	5.5162	5.5921	5.6684	5.7426
	*f*	5.1216	5.1220	5.1223	5.1230	5.1263	5.2007	5.2723	5.3442	5.4142
Me–O distances (Å)	*p*	2.0113	2.0056	1.9987	1.9923	1.9861	2.0543	2.1027	2.1523	2.1990
	*q*	1.9484	1.9588	1.9712	1.9829	1.9973	1.9579	1.9500	1.9405	1.9337
	*r*	3.7310	3.7508	3.7745	3.7970	3.8245	3.7491	3.7340	3.7158	3.7028
	*s*	3.6674	3.6711	3.6754	3.6797	3.6865	3.7171	3.7567	3.7959	3.8349
Bond angles (degree)	*θ*_1_	122.23	122.00	121.74	121.49	121.21	122.67	123.41	124.17	124.85
	*θ*_2_	140.27	139.39	138.35	137.40	136.35	142.08	145.27	148.86	152.38
	*θ*_3_	94.62	94.98	95.43	95.85	96.33	93.90	92.73	91.58	90.58
	*θ*_4_	126.30	126.38	126.47	126.56	126.66	126.14	125.89	125.63	125.40
	*θ*_5_	71.54	70.98	70.31	69.68	68.99	72.70	74.68	76.80	78.75

The tetrahedral and octahedral bond lengths (*d*_AL_) and (*d*_BL_), tetrahedral edge length (*d*_AE_), and shared octahedral edge lengths (*d*_BE_) and un-shared lengths (*d*_BEU_) have been calculated using eqn (A9)–(A13). The calculated *d*_AL_, *d*_BL_, *d*_AE_, *d*_BE,_ and *d*_BEU_ values for both the series have been listed in Table 3. From this Table, it is seen that the values of *d*_AL_, *d*_AE_ have increased while the values of *d*_BL_, *d*_BE,_ and *d*_BEU_ have reduced with an increase in Cr content for both the series S1, and S2.

Magnetic interaction strength for cubic spinel and spinel-like ferrite depends on the cation–cation (Me–Me) bond length and cation–anion (Me–O) bond length and bond angle that have been calculated using the eqn (A14)–(A27). All the calculated bond lengths and bond angles have been presented in Table 3. As seen in Table 3, there is decreasing and increasing trend in Me–Me for the S1 and S2 series, respectively. The Me–O distance shows an increasing trend for both the series. The bond angles show a decreasing trend with the increasing Cr content for both series. The decreasing trend of Me–Me and the bond angles may be due to the smaller ionic radius of Cr^3+^ than that of the Co^2+^ for the samples of the series S1. But in the case of the samples of series S2, this decreasing in Me–Me and bond angles is most likely due to the combined effects of smaller ionic radius of Cr^3+^ and excess of Co^2+^ ions therein. This behavior of bond length and bond angles for the samples of the series S1, and S2 imply the lattice expansion with the increase in Cr content, which is found to be in agreement with the lattice volume.

To confirm the presence of cation or anion vacancies, estimation of ionic packing coefficient is required. The ionic packing coefficient of tetrahedral site *P*_tet_ and octahedral site *P*_oct_ have been calculated by the eqn (A28) and (A29), respectively. The values for *P*_tet_ and *P*_oct_ have been presented in Table 4 for both the series. From Table 4, it is observed that the values of *P*_tet_ and *P*_oct_ are less than one, which suggests the existence of cation and anion vacancies in the parent COF.^34^ For series S1, the values of *P*_tet_ and *P*_oct_ increase for increasing Cr content and the values are close to 1, which indicates the reduction of ion (cation and anion) vacancies. On the other hand, *P*_tet_ and *P*_oct_ have been found to be decrease with increasing Cr^3+^ content which indicates enhancement of ion (cation and anion) vacancies.

**Table 4:** Oxygen positional parameter, ionic packing coefficient, fulfillment coefficient, vacancy parameter, and tolerance factor for all samples of series S1 (Co_1−*x*_Cr_*x*_Fe_2_O_4_) and S2 (Co_1+*x*_Cr_*x*_Fe_2−*x*_O_4_)

Series	*x*	Oxygen positional parameter, *u* and deviation, *δ*	Ionic packing coefficient			Fulfillment coefficient *α*	Vacancy parameter *β*	Tolerance factor *T*
		*u*	*δ*	*P*_a_	*P*_b_			
S1	0	0.3845	0.0095	0.9867	0.9967	0.5819	0.5145	1.038
	0.125	0.3852	0.0102	0.9877	0.9982	0.5811	0.4370	1.040
	0.250	0.3860	0.0110	0.9884	0.9988	0.5804	0.3783	1.043
	0.375	0.3868	0.0118	0.9919	0.9991	0.5782	0.0526	1.045
	0.500	0.3877	0.0127	0.9944	1.0008	0.5758	−0.1860	1.048
S2	0.125	0.3831	0.0081	0.9426	0.9589	0.5887	4.7671	1.027
	0.250	0.3807	0.0057	0.9016	0.9239	0.5972	8.5123	1.014
	0.375	0.3784	0.0033	0.8621	0.8940	0.6062	11.9433	1.000
	0.500	0.3763	0.0013	0.8259	0.8692	0.6150	14.9758	0.990

The degree of ionic packing coefficient can be evaluated by calculating the fulfillment coefficient (*α*) of the unit cell using eqn (A30). The vacancy parameter *β* is defined as normalized values of ions at the nodal point of the spinel structure which is calculated using eqn (A31). The values of *α* (tabulated in Table 4) show close to 0.58 for all the samples which confirm that our synthesized samples exhibit inverse spinel structure as explained in the earlier literature.^34^ The values of *β* listed in Table 4 show a low vacancy parameter which implies that the missing ions are lesser for parent COF. For series S1 the values of *β* shows the decreasing trend up to *x* = 0.375 that implies reducing the missing ions due to Cr substitution. But at higher Cr content, it shows the negative value, which are most likely due to the excess of ions. The excess of ions dominates due to higher Cr content in series S1. But in series S2, *β* values increase strongly with an increase of Cr content that dominates the enhancement of metallic behavior from semiconducting behavior due to the higher values of Co^2+^ ions.^35^

The tolerance factor (*T*) is another property to get an idea about the impurities of these type of materials. Hence the *T* have been calculated using eqn (A32) that have been tabulated in Table 4 for both the series. It is observed that *T* is slightly higher than one which indicates that the synthesized sample is slightly distorted from the inverse spinel structure. In both the series, the values of *T* are found to decrease with the increase of Cr content indicating the reduction of distortion from the central atom resulting the improvement of inverse spinel structure.

#### 3.1.5 Crystallite size estimation

To evaluate crystallite size, analysis of X-ray profiles is the most effective and easiest way. To calculate crystallite size and strain of the powdered samples various methods have been used such as: Scherrer, Modified Scherrer, Size strain plot, and Williamson–Hall plot. In every case, XRD data have generally been used because of X-ray line broadening comes out mainly from three factors: (i) instrumental effect, (ii) crystallite size, and (iii) local lattice strain. To exclude the instrumental broadening, a standard silicon X-ray powder diffraction data is recorded under the same condition and eliminated from the observed peak width. The full width at half maximum (FWHM) of all peak positions from the XRD has been estimated using a nonlinear combined curve fitting function that includes Gaussian and Lorentzian functions. The FWHM data has been calculated by using the eqn (A33). In addition, the instrumental broadening (*β*_i_) is removed by using the eqn (A34). The values for *β*_i_ are 0.092°, 0.099°, 0.117°, 0.186°, 0.21°, and 0.208° for the (220), (311), (400), (511), and (440) peaks, respectively. The average crystallite size (*D*) have been calculated by using observed FWHM of the most intense peak (311) with the help of Scherrer equation (eqn (A35)). The calculated values of *D* using this method are listed in Table 5.

**Table 5:** Crystallite size (*D*) and strain (*ε*) estimated by different methods for all samples of series S1 (Co_1−*x*_Cr_*x*_Fe_2_O_4_) and S2 (Co_1+*x*_Cr_*x*_Fe_2−*x*_O_4_)

Series	*x*	Scherrer method	Modified Scherrer method	Rietveld refined method		Williamson–Hall Method		Size Strain Method	
		*D* (nm)	*D* (nm)	*D* (nm)	*ε*	*D* (nm)	*ε*	*D* (nm)	*ε*
S1	0	46	53	61	0.00092	71	0.000725	60	1.41 × 10^−5^
	0.125	46	51	57	0.00085	70	0.000746	52	5.91 × 10^−6^
	0.250	45	49	52	0.00071	64	0.000775	51	9.35 × 10^−6^
	0.375	39	45	51	0.00058	60	0.000909	50	1.33 × 10^−5^
	0.500	39	43	46	0.00046	56	0.000905	47	1.40 × 10^−5^
S2	0.125	47	50	59	0.00075	67	0.000786	58	1.14 × 10^−5^
	0.250	40	43	49	0.00068	52	0.000624	45	5.94 × 10^−5^
	0.375	37	40	41	0.00051	43	0.000374	42	5.55 × 10^−6^
	0.500	39	39	38	0.00046	43	0.000353	40	2.21 × 10^−6^

Scherrer equation is used to determine *D* for most intense peak, but since the constant value of cos *θ* is not maintained with the increase in 2*θ* and resulting errors in the *D* values. To avoid the above-mentioned errors, Monshi *et al.* in 2012 employed some modifications in the use of the Scherrer equation and introduced the following formula:^18^
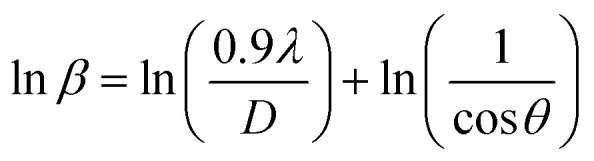


**Fig. 8:**
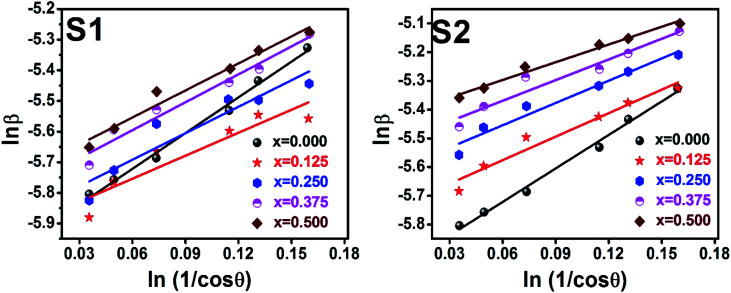
Modified Scherer plots for all samples of series S1 (Co_1−*x*_Cr_*x*_Fe_2_O_4_) and S2 (Co_1+*x*_Cr_*x*_Fe_2−*x*_O_4_).

To evaluate the more accurate values of *D*, 
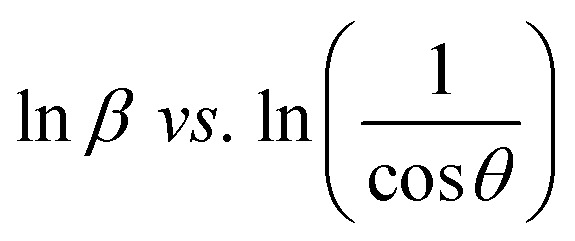
 the graph has been plotted for all the selected peaks. Then *y*-intercepts, 
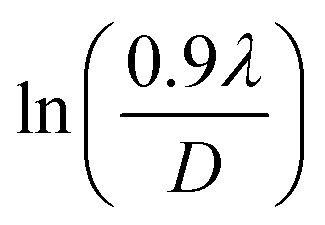
, have been determined by the linear fitting on this graph using the origin software for the sample of series S1, and S2, as shown in Fig. 8. The calculated *D* values taking 
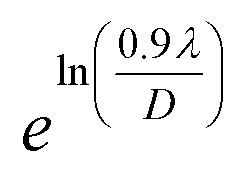
 for all the samples have been tabulated Table.

**Fig. 9:**
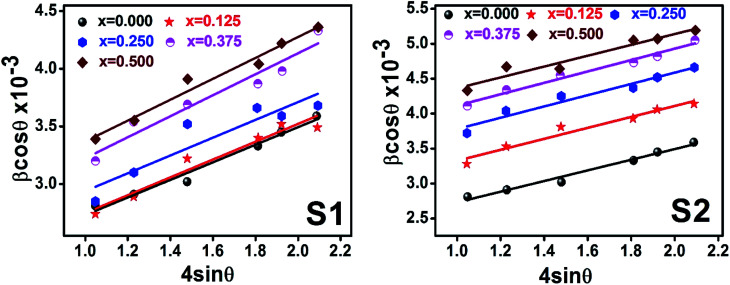
Williamson–Hall Plot for all samples of series S1 (Co_1−*x*_Cr_*x*_Fe_2_O_4_) and S2 (Co_1+*x*_Cr_*x*_Fe_2−*x*_O_4_).

Scherrer and modified Scherrer methods can provide the only information about the crystallite size but not the information about the intrinsic strain of the lattice. As such, Williamson–Hall method has been utilized to calculate both *D* and the strain, *ε* of the materials. According to this method, a total physical broadening, *β*_tot_, have been calculated by the following relation:^14,17^
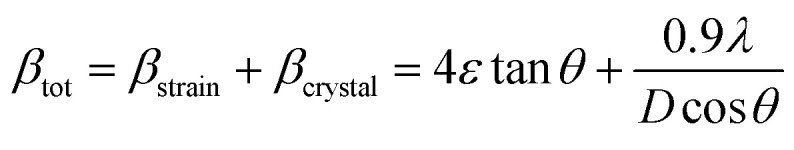
or,
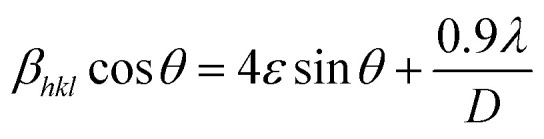
Here, *β*_*hkl*_ is the total broadening due to strain and size in a particular peak having the (*hkl*) value which is written exchange of *β*_tot_. Fig. 9 show the *β*_*hkl*_ cos *θ vs.* 4 sin *θ* graph corresponding to each diffraction peak for the samples of series S1, and S2, respectively. In every case for all the samples, the slope and *y*-intercept have been noted from the linear fitted curve, which gives the *ε* and *D* of the investigated samples. The *ε* and *D* for all the samples have been listed in Table 5 in Appendix B.

**Fig. 10:**
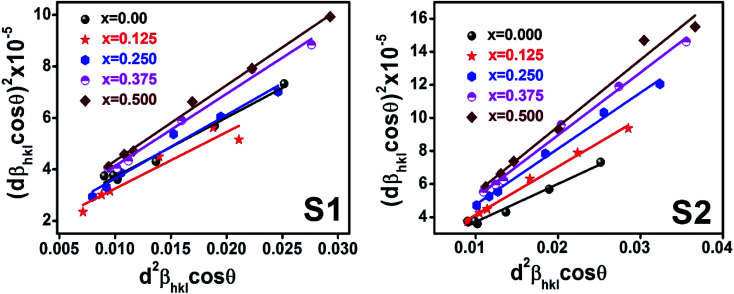
Size strain plot for all samples of series S1 (Co_1−*x*_Cr_*x*_Fe_2_O_4_) and S2 (Co_1+*x*_Cr_*x*_Fe_2−*x*_O_4_).

**Fig. 11:**
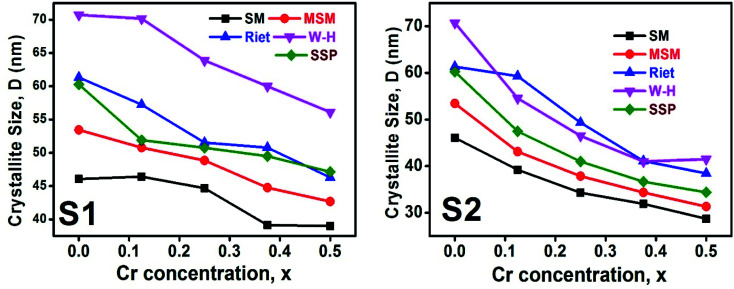
The *D* values calculated using Scherer method (SM), modified Scherer method (MSM), Rietveld method (riet), Williamson–Hall (W–H), and size strain plot (SSP) for all samples of series S1 (Co_1−*x*_Cr_*x*_Fe_2_O_4_) and S2 (Co_1+*x*_Cr_*x*_Fe_2−*x*_O_4_).

Williamson–Hall method describes isotropic peak broadening due to the combination of size and strain-induced effect as a function of a 2*θ*.To better evaluation of *ε* and *D* another model known as “size–strain plot (SSP)” has been used. In this method, higher angle reflections are less important than lower angle reflections. In the SSP method, the following relation has been considered:^17^
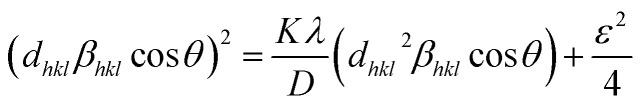
Here, *d*_*hkl*_ is lattice spacing for different (*hkl*) planes. The (*d*_*hkl*_*β*_*hkl*_ cos *θ*)^2^ are plotted as a function of *d*_*hkl*_^2^*β*_*hkl*_ cos *θ* and their linear fitted curve have been drawn using the origin software as shown in Fig. 10. The slope and *y*-intercept have been extracted for all the samples that provides the values of *D* and *ε* of both series and presented in Table 5. In addition, the *D* and *ε* values extracted by the Match-3 software from the Rietveld refined XRD data have also been listed in Table 5. The *D* values calculated by using all the methods is found to be decreasing trend with the increasing Cr content for both series as shown in Fig. 11. This decreasing trend is most likely due to the peak broadening.

### 3.2 FTIR analysis

**Fig. 12:**
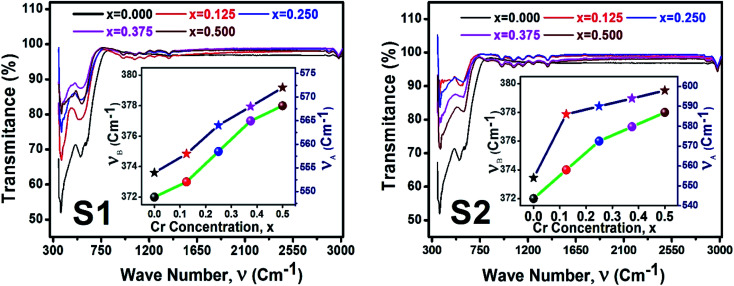
FTIR spectrum for all samples of series S1 (Co_1−*x*_Cr_*x*_Fe_2_O_4_) and S2 (Co_1+*x*_Cr_*x*_Fe_2−*x*_O_4_).

Fig. 12 illustrate the FTIR spectrum in the wavenumber range of 350–3000 cm^−1^ for the samples of both series at room temperature. From the FTIR spectra, two distinct absorption bands at 554 cm^−1^ (*ν*_1_) and 372 cm^−1^ (*ν*_2_) are observed for the parent COF. The symmetrical stretching vibrations of metal–oxygen at tetrahedral and octahedral sites have been observed at the higher band *ν*_1,_ and lower band *ν*_2_, respectively. For all samples, it is seen that both *ν*_1,_ and *ν*_2_ are increased with the increase of Cr content as shown in the inset of Fig. 12. The values *ν*_1,_ and *ν*_2_ have been listed in Table 6. A clear absorption band has been noticed at around 3000 cm^−1^ (*ν*_3_). This may be attributed to the H–O–H stretching vibrations due to the effect of moisture during fabrication of the studied samples.

**Table 6:** Absorbtion wave number (*ν*_1_, *ν*_2_), force constant, stiffness constant, wave velocity, elastic constant, Pugh ratio, Poisson ratio Debye temperature, and minimum thermal constant for all samples of series S1 (Co_1−*x*_Cr_*x*_Fe_2_O_4_) and S2 (Co_1+*x*_Cr_*x*_Fe_2−*x*_O_4_)

Series			S1					S2			
x			0	0.125	0.250	0.375	0.500	0.125	0.250	0.375	0.500
*ν*_1_ (cm^−1^)			371	373	375	377	378	374	376	377	378
*ν*_2_ (cm^−1^)			554	558	564	568	572	586	590	594	598
Force constant	*k*_t_ (N m^−1^)		290	294	300	305	309	324	329	333	338
	*k*_o_ (N m^−1^)		130	131	133	134	135	132	133	134	135
	*k*_ab_ (N m^−1^)		210	213	217	219	222	228	231	234	236
Stiffness costant	*C*_11_ (GPa)		251	255	260	264	268	273	277	280	284
	*C*_12_ (GPa)		105	106	106	106	106	110	110	109	108
Wave velocity	*v*_l_ (ms^−2^)		6843	6911	6999	7080	7158	7035	6986	6940	6912
	*v*_t_ (ms^−2^)		3950	3990	4041	4087	4133	4062	4033	4007	3991
	*v*_m_ (ms^−2^)		4156	4197	4250	4299	4348	4272	4243	4215	4198
Elastic constant	*B* (GPa)		153	156	157	158	160	164	165	166	167
	*G* (Gpa)		84	84	87	88	89	91	92	93	95
	*E* (GPa)		216	220	223	226	229	234	237	239	241
Pugh ratio (*B*/*G*)			1.84	1.83	1.82	1.80	1.79	1.81	1.79	1.78	1.76
*Σ*			0.29	0.29	0.29	0.29	0.28	0.29	0.28	0.28	0.28
Zero porosity elastic constant		*E*_0_ (GPa)	261	266	280	291	300	298	310	322	340
		*G*_0_ (Gpa)	100	102	107	111	115	114	119	124	131
		*B*_0_ (GPa)	226	230	238	244	250	252	259	265	275
*σ*_0_			0.31	0.31	0.30	0.30	0.30	0.30	0.30	0.29	0.29
*B*_0_/*G*_0_			2.26	2.25	2.22	2.19	2.16	2.20	2.16	2.14	2.09
*θ*_D_ (K)			593	599	606	612	618	610	605	601	598
*K*_min_			1.31	1.33	1.34	1.36	1.37	1.35	1.34	1.33	1.32

#### 3.2.1. Calculation of force constant

**Fig. 13:**
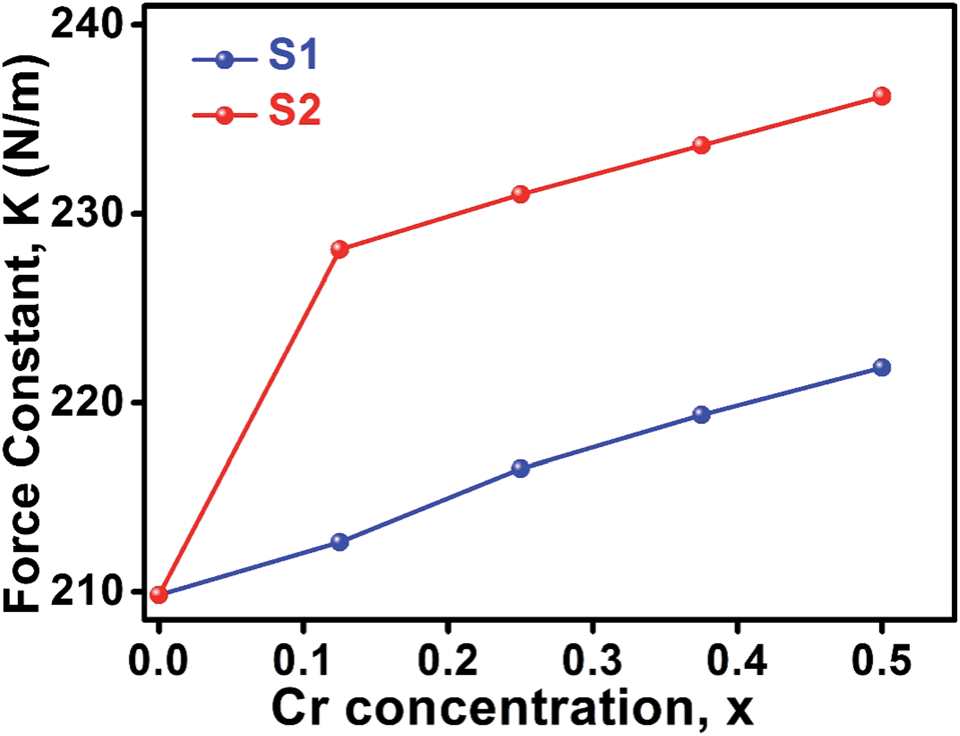
Force constant as a function of Cr content all samples of series S1 (Co_1−*x*_Cr_*x*_Fe_2_O_4_) and S2 (Co_1+*x*_Cr_*x*_Fe_2−*x*_O_4_).

The wavenumbers *ν*_1_ and *ν*_2_ of the infrared active phonon mode is directly connected to the force constant. The force constant *k*_t_ and *k*_o_ at the tetrahedral and octahedral site of cubic spinel structure has been calculated by the Waldron relation (eqn (A39)–(A41)). The average force constant *k*_av_ = (*k*_t_ + *k*_o_)/2 have shown in Fig. 13 for the all samples of both series. From the Fig. 13, it is seen that the average force constant *k*_av_ increases for the samples of both series which is as usually related to the Me–Me bond distances and bond angles.

#### 3.2.2 Elastic properties analysis

Ferrites demonstrate important elastic properties and thermal behavior due to their interatomic and interionic forces. Although the elastic properties of such materials are determined by applying external stress, according to the analysis of various research, a technique based on the structural and FTIR data parameter related to elastic properties along with thermal properties has been calculated for exploring the correlations with the other properties.^1,20–22^ According to Hook's law, the stress *η*_*i*_, strain *ε*_*ij*_and stiffness *C*_*ij*_ are correlated on the basis of the stress–strain approach.^40^ The stiffness *C*_*ij*_ are used to calculate the elastic constants. For cubic symmetry, only three stiffness *C*_11_, *C*_12_, and *C*_44_ are considered to be dominant, where *C*_11_ represents the elasticity in length and *C*_12_ and *C*_44_ represents the elasticity in shape. The stiffness constant *C*_11_ and *C*_12_ are calculated using eqn (A43) and (A44), and the values are tabulated in Table 6.

**Fig. 14:**
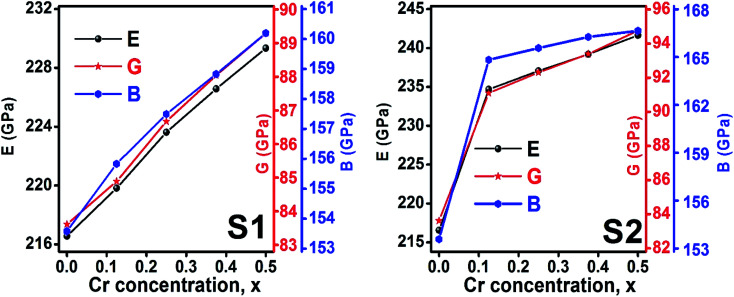
Elastic modulus as a function of Cr content for all samples of series S1 (Co_1−*x*_Cr_*x*_Fe_2_O_4_) and S2 (Co_1+*x*_Cr_*x*_Fe_2−*x*_O_4_).

The bulk modulus, *B*, rigidity modulus, *G*, Young's modulus, *E*, Poisson's ratio, *σ*, longitudinal wave velocity, *v*_l_, transverse wave velocity, *v*_t_, and the mean velocity, *v*_m_, have also been calculated for all samples of both series using the eqn (A46)–(A51). The calculated values of all these elastic constants with Cr content for both the series have been graphically presented in Fig. 14. The values of *E*, *B*, and *G* are found to increase with increasing Cr content. The measured elastic moduli however do not provide enough information on the mechanical properties of the investigated samples due to the presence of porosity. Hence, to improve the elastic nature of the materials, the corrected zero porosity elastic moduli have been calculated by using Hasselman and Fularth's formula (eqn (A51)–(A54)). The corrected zero porosity elastic moduli (*E*_0_, *B*_0_, and *G*_0_) have been listed in Table 6 which shows the larger value than the measured *E*, *B*, and *G*. From Table 6, it is seen that the values of *E*_0_, *B*_0_, and *G*_0_ show the similar trend with the values of *E*, *B*, and *G* as a function of Cr concentration for the samples of both series.

**Fig. 15:**
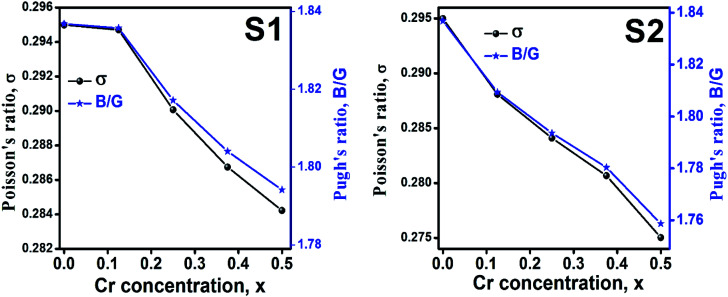
Poison's ratio and Pugh's ratio as a function of Cr content for all samples of series S1 (Co_1−*x*_Cr_*x*_Fe_2_O_4_) and S2 (Co_1+*x*_Cr_*x*_Fe_2−*x*_O_4_).

Ductility and brittleness behavior of a material can be estimated by the Pugh's ratio.^42^ The Pugh's ratios (B/G) are illustrated in Fig. 15 for the samples of both series. According to Frabtsevich *et al.*^43^ the brittleness and ductility nature can be confirmed from the value of *σ*. The calculated values of *σ* are also illustrated in Fig. 15. It is observed that both the Pugh's ratio and *σ* are higher than that of their respective critical values 1.75 and 0.26, respectively. This reveals the ductile nature of the synthesized samples.^42,43^ The ductility decrease with the increase of Cr content for both the series which may be due to the substitution of brittle Cr with ductile Co and Fe for the sample of both series. In addition, *σ* for all the samples are in the range of 0.27–0.30 which lies in between −1 to 0.5 which implies that the investigated samples are of isotropic elastic in nature.

#### 3.2.3 Thermodynamic properties

**Fig. 16:**
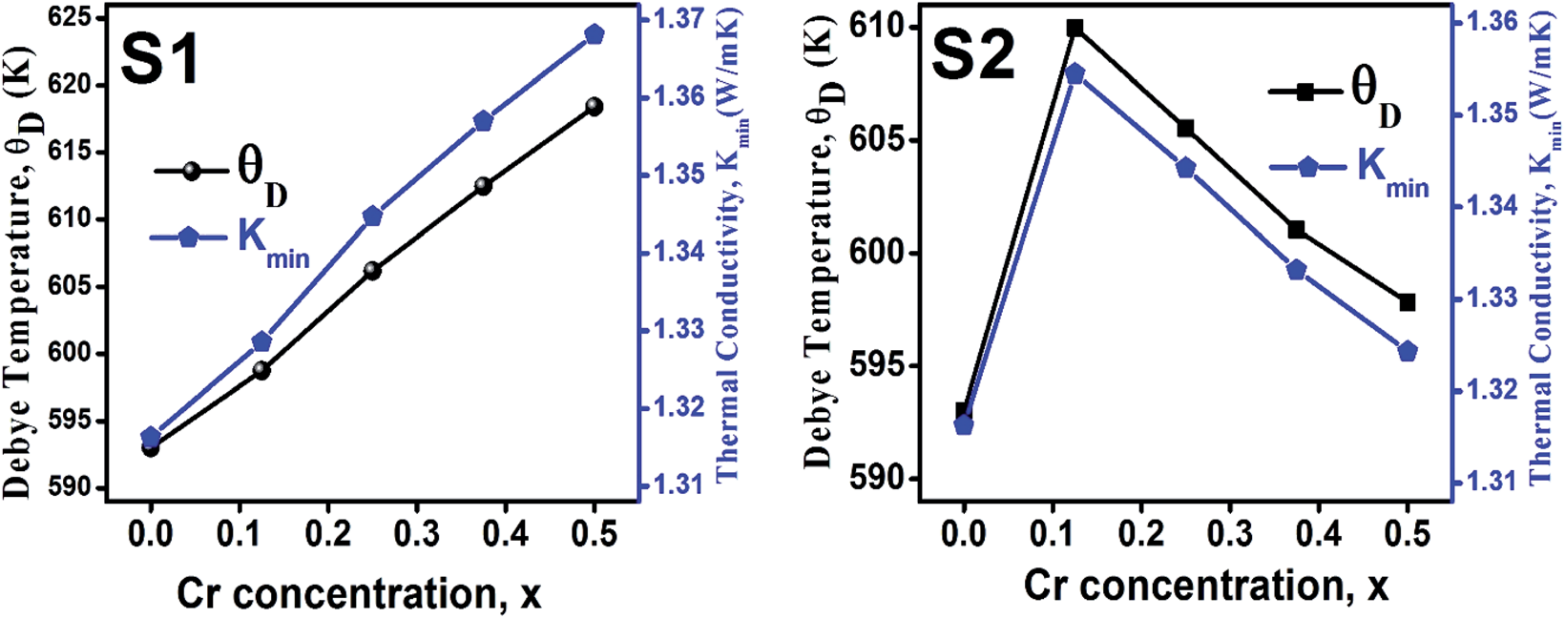
Debye temperature and thermal conductivity as a function of Cr content for all samples of series S1 (Co_1−*x*_Cr_*x*_Fe_2_O_4_) and S2 (Co_1+*x*_Cr_*x*_Fe_2−*x*_O_4_).

The Debye temperature, *θ*_D_, is a significant parameter to know about the thermodynamic properties of a solid that originates from the maximum lattice vibration of the atoms. According to Anderson's formula the *θ*_D_ can be calculated using the eqn (A55). Thermal conductivity is one of the thermodynamic properties of a material that indicates the ability to conduct heat. The minimum value of thermal conductivity, *K*_min_, have been calculated by the eqn (A56). The calculated *θ*_D_ and *K*_min_ have been presented in Fig. 16 as a function of Cr content for all samples of both series. It is observed that *θ*_D_ and *K*_min_ increases with the increase of Cr content for S1 series. However, for S2 series both *θ*_D_ and *K*_min_ shows the maximum value for *x* = 0.125, and beyond this value of Cr content they decrease.

## 4. Discussion

The analysis of structural, mechanical, and thermal properties of the samples of two series S1, and S2, show almost similar behavior in most of their properties. For the stoichiometric series S1, the XRD pattern shows a single phase cubic structure for all Cr contents. For a lower value of Cr^3+^ substitution with Co^2+^, cation–anion vacancies dominated. However, for higher values of Cr^3+^, negative values of vacancy parameter have been found. This negative vacancy parameter dominates the excess Cr^3+^ replaced with the Co^2+^ and these excess ions are not too high, so no impurity peaks have been observed in the XRD patterns. A slight distortion of inverse spinel structure has been confirmed from the tolerance factor for all samples of both series. The dominance of Cr^3+^ with extra Co^2+^ ions due to lowering the packing fraction demonstrates strongly increasing trend of vacancy parameter with increase of Cr^3+^ and leading to transform semiconducting to metallic behavior in S2 series. However, a single phase cubic structure is observed for the samples of S2 series without any impurity peak in the XRD patterns. The observed anion displacement for the samples of two series are dominated by the deviation of oxygen positional parameters from their standard value of 0.375. Expansion of lattice volume for the series S1 has been observed due to the larger ratio of Cr^2+^/Co^2+^ at tetrahedral site than the Cr^3+^/Co^2+^ ratio at octahedral site. In S2 series, Fe^3+^ has been replaced by Cr^3+^ with a simultaneous addition of Co^2+^, resulting an increase in lattice volume. In series S1, both the *d*_x_ and *d*_B_ show a decreasing trend, which may be attributed to the increase of Cr^3+^ with a lower density of 7.14 g cm^−3^ than that of Co^2+^ (8.9 g cm^−3^).^45^ Although pelletization pressure and sintering temperature for all samples were the same, there is an increasing trend of ductility with an increase of Cr content. At higher sintering temperature (>1100 °C) the ductility of Cr/Cr-based alloys and composite is reported to improve.^46^

In series S2, Cr metal having lower density replaced the Fe (7.87 g cm^−3^) with slightly higher density.^45^ However, addition of same amount of Co metal with high density, resulted in the increasing trend of *d*_B_, and *d*_x_ with a increase in porosity. However, the ductility is found to decrease possibly due to excess Co^2+^. In addition, an increase of excess Co with lower shear modulus presumably plays a prominent role in lowering the ductile nature for the sample series S2. The decreasing trend of ductility as a function of Cr content are observed due to the increase of the Cr^3+^/Fe^2+^ ratio. In series S1, the *θ*_D_ shows an increasing trend due to the greater *θ*_D_ of Cr than that of Co.^47^ In series S2, the *θ*_D_ increases at lower Cr content (=0.125) but at a higher Cr content (>0.125), *θ*_D_ decreases due to the increase of Co/Cr ratio, and decrease of Fe/Cr ratio.

## 5. Conclusions

Two series of Cr^3+^ substituted cobalt ferrite with stoichiometric (S1 series) and non-stoichiometric (S2 series) ratios have been synthesized by the standard solid-state reaction technique. XRD patterns for all samples of both series indicate a single phase cubic spinel structure with a space group of *Fd*3̄*m*. FTIR spectra of the synthesized samples also confirm the spinel structure. The cation distribution calculated from the extracted Rietveld refined data confirms the mixed spinel structure of all samples of both series. In both the stoichiometric and non-stoichiometric series, an enhancement of lattice parameter have been found. From the analysis of elastic properties, it is found that all the samples for both series are ductile in nature. For the stoichiometric series Cr^3+^ together with Co^2+^ is assumed to have influenced the structural and mechanical properties. In addition, non-stoichiometric compositions provide an opportunity to tailor Co^2+^/Cr^3+^ and Fe^3+^/Cr^3+^ in the inverse spinel ferrite which causes the defect in structure, and modification of mechanical behavior.

## 6 Appendix-A

Equations belonging to Section 3:

Equation for theoretical lattice parameter:^1^A1
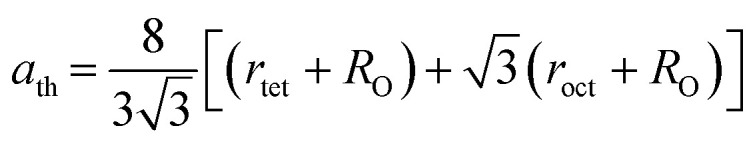
where *R*_O_ (=1.32 Å) is the ionic radius of oxygen.

The N–R function is as follows:^17^A2
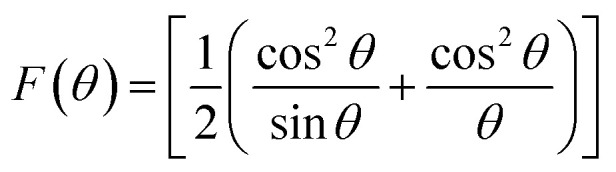


The lattice parameter for each Bragg position:^30^A3
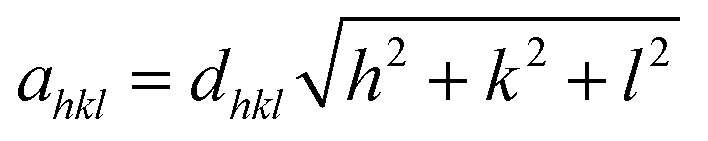
Here, *a*_*hkl*_, *d*_*hkl*_ are the lattice constant and interplanar spacing respectively.

Equations for bulk density *d*_B_and X-ray density *d*_x_^32^A4
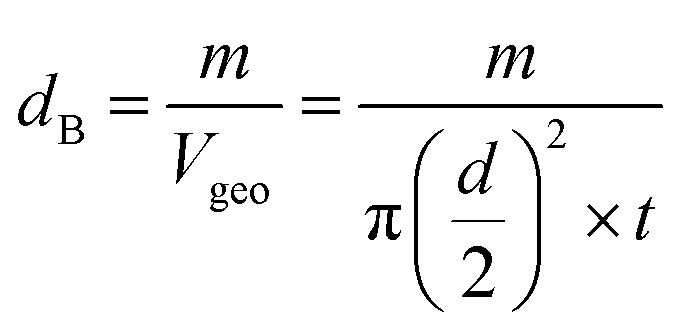
A5
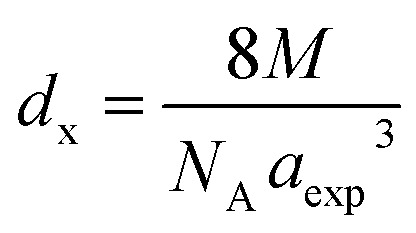


Equation for porosity:^8^A6
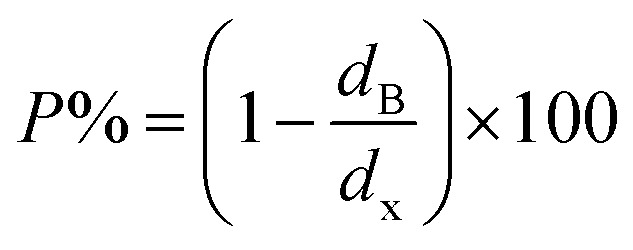
where *M* is the molecular mass calculated from molecular formulae, *a*_exp_ is the experimental lattice constant and *N*_A_ (=6.02 × 10^23^) is Avogadro's number.

Equation for oxygen positional parameter *u*:^1^A7
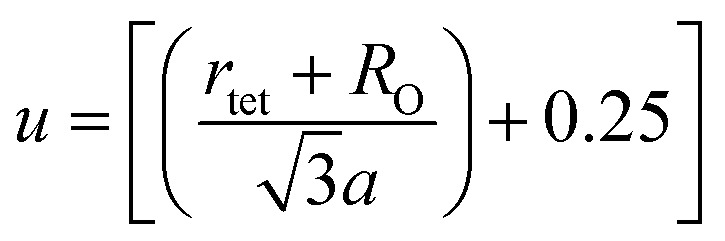


Stanley's equations:^1^A8
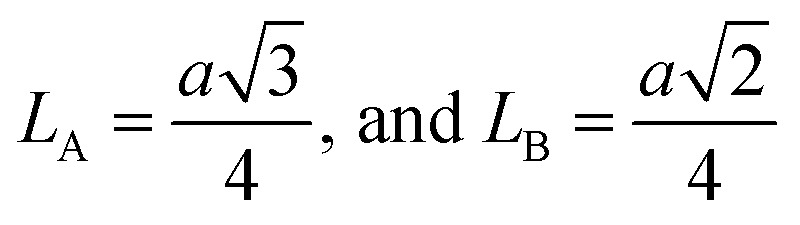


The equations for tetrahedral and octahedral bond lengths (*d*_AL_) and (*d*_BL_), tetrahedral edge length (*d*_AE_), and shared (*d*_BE_) and un-shared octahedral edge lengths (*d*_BEU_) are as follows:^1^A9
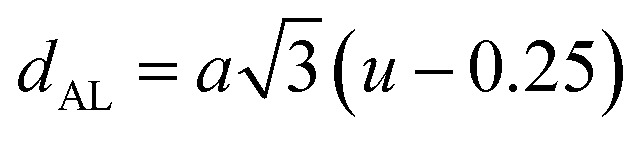
A10
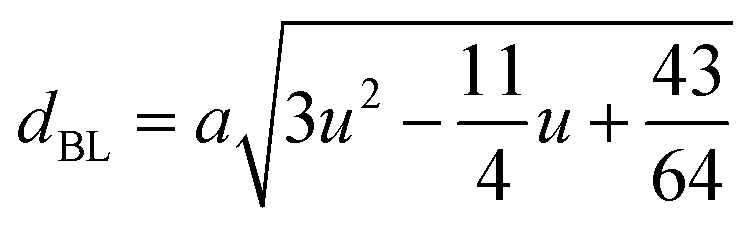
A11
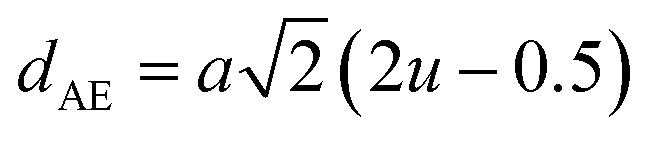
A12
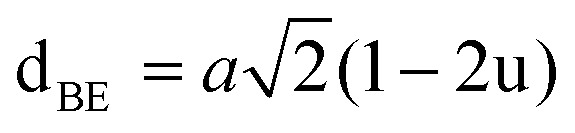
A13
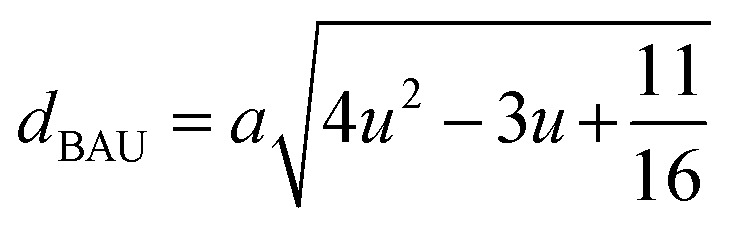


Equations for various bond length and bond angles:^1^

Me–Me distancesA14
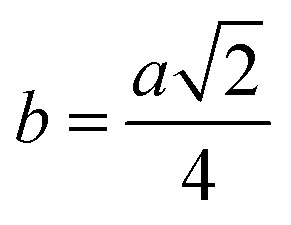
A15
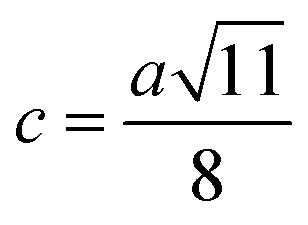
A16
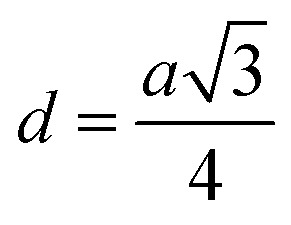
A17
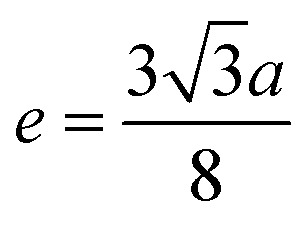
A18
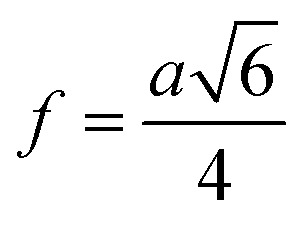


Me–O distancesA19
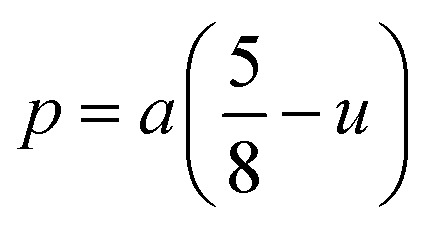
A20
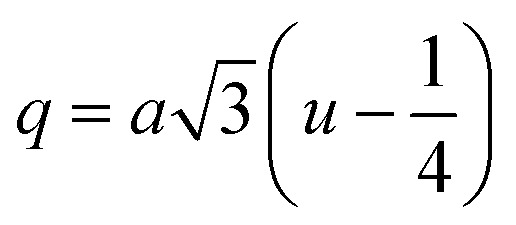
A21
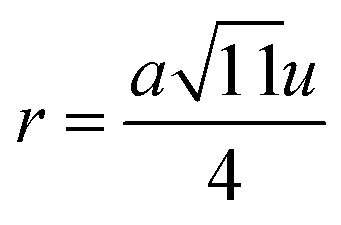
A22
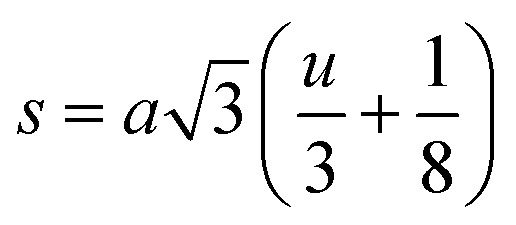


Bond anglesA23
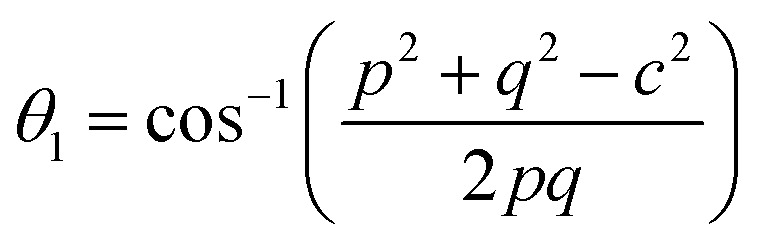
A24
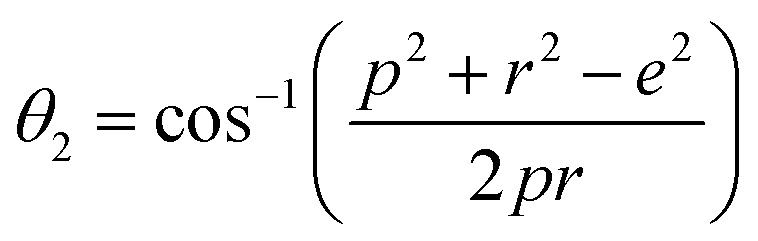
A25
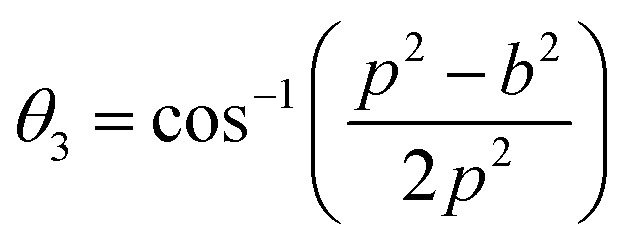
A26
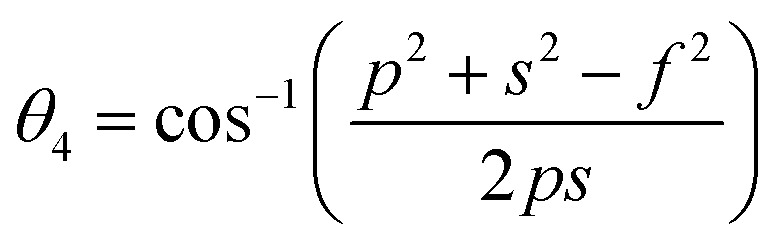
A27
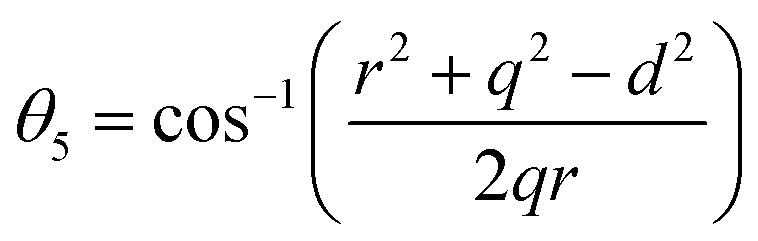


Equations for the ionic packing coefficient *P*_tet_ and *P*_oct_ at the tetrahedral and octahedral sites:^34^A28
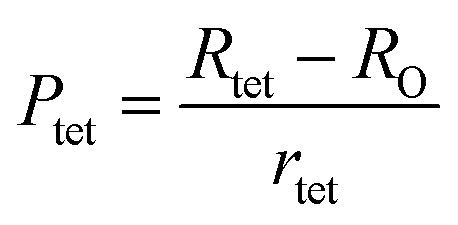
A29
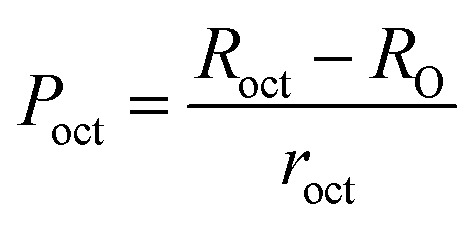
Here, *R*_tet_ (=*d*_AL_) and *R*_tet_ (=*d*_BL_)are the average bond length at tetrahedral and octahedral sites respectively.

Equation for the fulfillment coefficient (*α*) of the unit cell using the following relation:^34^A30
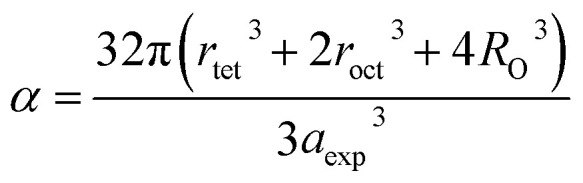


Equation of vacancy parameter *β*:^34^A31
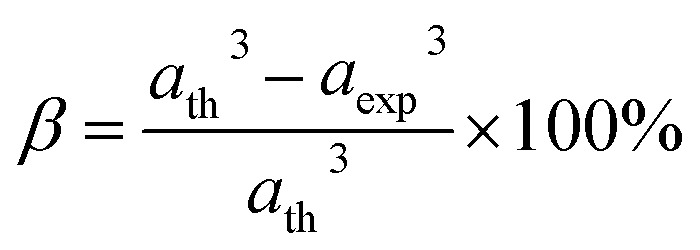


Equation for tolerance factor calculation:^36^A32
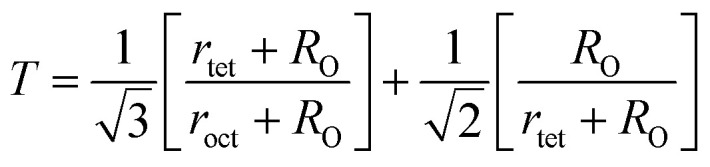


The FWHM data has been calculated by using the following relation:^37^A33
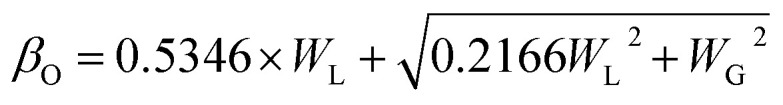
Here, *β*_O_ is observed FWHM, *W*_L_, and *W*_G_ is the Lorentzian and Gaussian width respectively. The instrumental broadening (*β*_i_) removal equation:^34^

A34 *β*_*hkl*_ = [(*β*_O_ − *β*_i_)(*β*_O_^2^ − *β*_O_^2^)^0.5^]^0.5^

The Scherrer equation:^17^A35
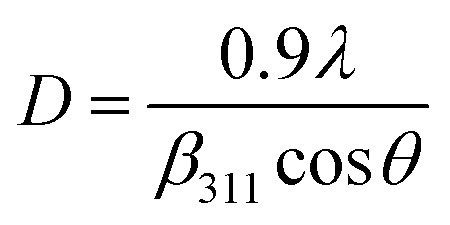
Here, *β*_311_ is the FWHM at (311) plane (most intense peak), *λ* (=1.5406 Å) represents the wavelength of incident X-rays.

Modified Scherrer equation:^22^A36
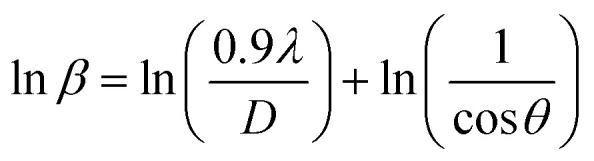


Williamson–Hall relation:^14,21^
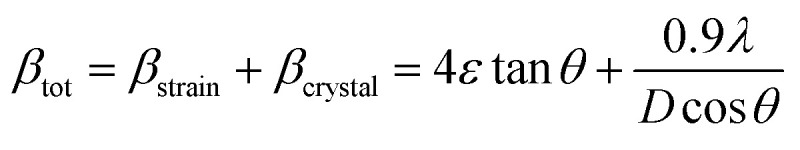
or,A37
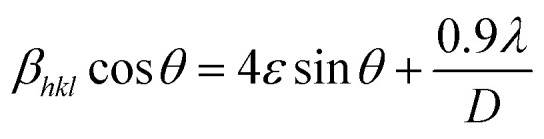
Here, *β*_*hkl*_ is the total broadening due to strain and size in a particular peak having the (*hkl*) value which is written exchange of *β*_tot_.

Equation for the size strain plot:^21^A38
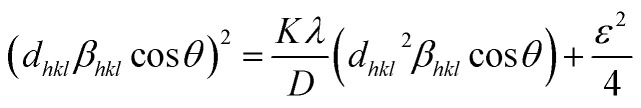
Here, *d*_*hkl*_ is lattice spacing for different (*hkl*) planes.

Force constant according to Waldron relation:^21^

A39 *k*_t_ = 4π^2^*c*^2^*ν*_1_^2^*μ*

A40 *k*_o_ = 4π^2^*c*^2^*ν*_2_^2^*μ*

A41 *k*_av_ = (*k*_t_ + *k*_o_)/2

Here, *c* is the velocity of light, *μ* is the reduced mass which we have calculated by the following relation:^38,39^A42
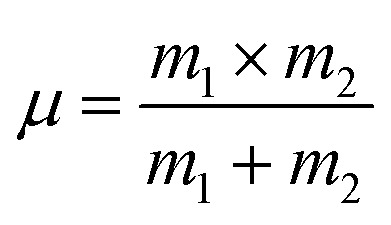
where *m*_1_ is the weighted average of atomic weights of the cations residing at the A-site or B-site while *m*_2_ is the atomic weight of oxygen anion.

Equation for the stiffness constant *C*_11_, *C*_12_:^21^A43
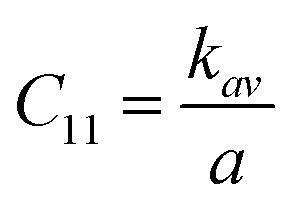
A44
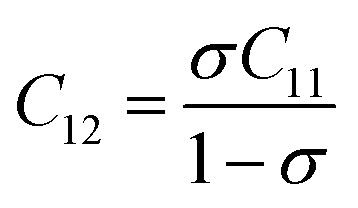
Here, *σ* is the Poisson's ratio as a function of pore fraction *P*. The corresponding relation for *σ* is as follows:^21^

A45 *σ* = 0.324(1 − 1.043*P*)

Some elastic properties related equations:^40,41^

Bulk modulus:A46
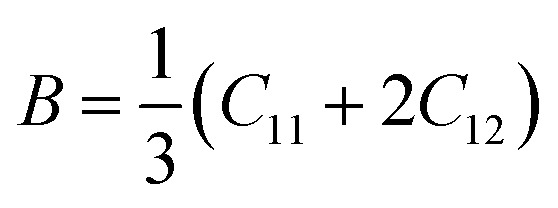


Longitudinal wave velocity,A47
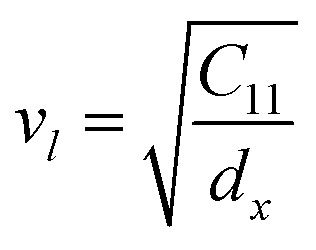
Here, *d*_x_ is the X-ray density.

Transverse wave velocity,A48
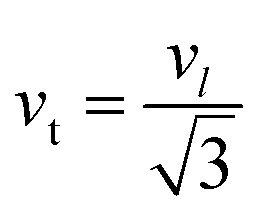


Rigidity modulus,

A49 *G* = *d*_x_*v*_t_^2^

Young's modulus,

A50 *E* = 2(1 + *σ*)*G*

Mean velocity,A51
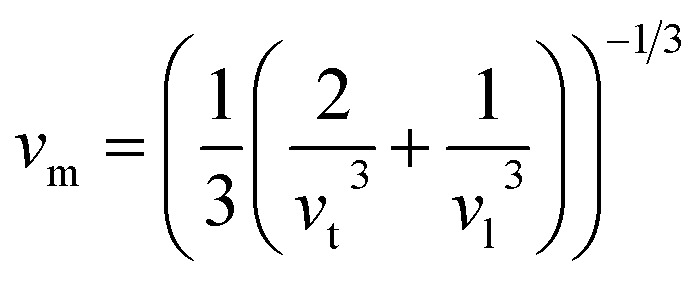


Hasselman and Fularth's formulae for the corrected zero porosity elastic moduli:^21,41^A52
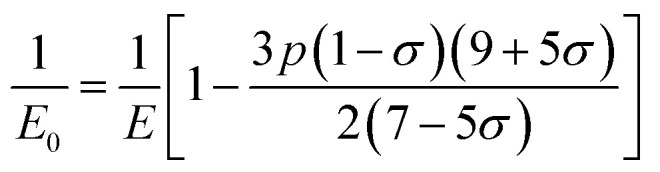
A53
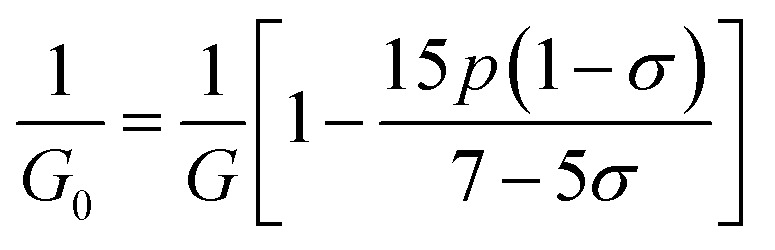
A54
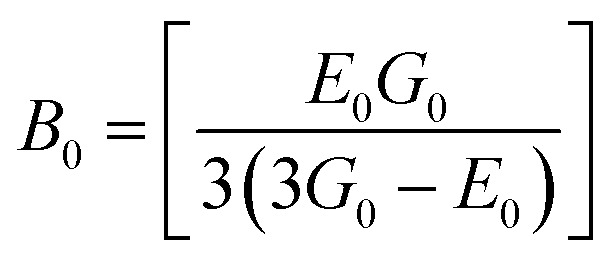
A55
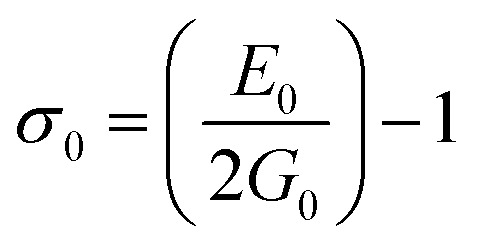


Anderson's formula for the Debye temperature, *θ*_D_:^44^A56
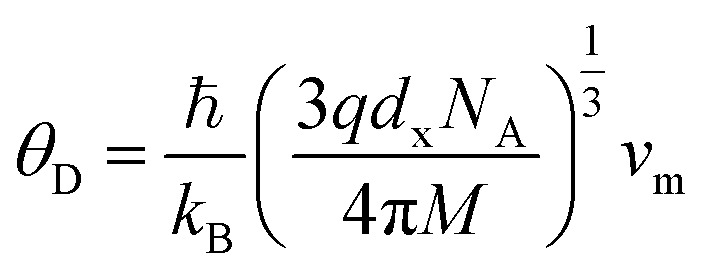
where ℏ is Planck's constant, *k*_B_ is Boltzmann's constant, *N*_A_ is Avogadro's number, *d*_x_ is the density, *M* is the molecular mass, and *q* is the number of atoms per unit formula.

Equations for thermal conductivity:^38^A57
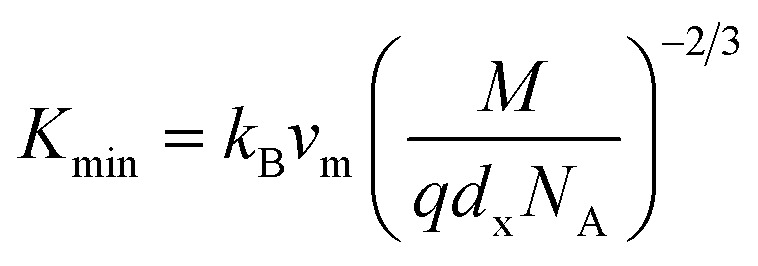


## 7 Appendix B

## Conflicts of interest

There is no conflicts to declare.

## Supplementary Material

RA-012-D1RA09090A-s001
