# Supplementary material for: Structural characteristics, cation distribution, and elastic properties of Cr3+ substituted stoichiometric and non-stoichiometric cobalt ferrites
Source: RSC Adv. 2022 Mar 17;12(14):8502–19. doi: 10.1039/d1ra09090a (PMC8985153; doi:10.1039/d1ra09090a)
Supplement: RA-012-D1RA09090A-s001 [file RA-012-D1RA09090A-s001.pdf]

This journal is © The Royal Society of Chemistry 2022

Appendix-A

Equations belonging to section 3:

Equation for theoretical lattice parameter [1]:

$$a_{th} = \frac{8}{3\sqrt{3}}\left[\left(r_{tet} + R_O\right) + \sqrt{3}\left(r_{oct} + R_O\right)\right] \cdots\cdots\cdots \quad \textbf{(A1)}$$

Where R_O (=1.32Å) is the ionic radius of Oxygen.

The N-R function is as follows [17]:

$$F(\theta) = \left[\frac{1}{2}\left(\frac{\cos^2\theta}{\sin\theta} + \frac{\cos^2\theta}{\theta}\right)\right] \cdots\cdots\cdots \quad \textbf{(A2)}$$

The lattice parameter for each Bragg position [30]:

$$a_{hkl,} = d_{hkl}\sqrt{h^2 + k^2 + l^2} \cdots\cdots\cdots \quad \textbf{(A3)}$$

Here, $a_{hkl,}\ d_{hkl}$ are the lattice constant and interplanar spacing respectively.

Equations for bulk density d_B and X-ray density d_x [32]

$$d_B = \frac{m}{V_{geo}} = \frac{m}{\pi\left(\frac{d}{2}\right)^2 \times t} \cdots\cdots\cdots \quad \textbf{(A4)}$$

$$d_x = \frac{8M}{N_A {a_{exp}}^3} \cdots\cdots\cdots \quad \textbf{(A5)}$$

Equation for porosity [8]:

$$\%P = \left(1 - \frac{d_B}{d_x}\right) \times 100 \cdots\cdots\cdots \quad \textbf{(A6)}$$

Where M is the molecular mass calculated from molecular formulae, a_{exp} is the experimental lattice constant and $N_A(= 6.02 \times 10^{23})$ is Avogadro's number.

Equation for oxygen positional parameter u [1]:

$$u = \left[\left(\frac{r_{tet} + R_O}{\sqrt{3}a} \right) + 0.25 \right] \cdots\cdots\cdots \textbf{(A7)}$$

Stanley's equations [1]:

$$L_A = \frac{a\sqrt{3}}{4}, and\ \ L_B = \frac{a\sqrt{2}}{4} \cdots\cdots\cdots \textbf{(A8)}$$

The equations for tetrahedral and octahedral bond lengths (d_{AL}) and (d_{BL}), tetrahedral edge length (d_{AE}), and shared (d_{BE}) and un-shared octahedral edge lengths (d_{BEU}) are as follows [1]:

$$d_{AL} = a\sqrt{3}(u - 0.25) \cdots\cdots\cdots \textbf{(A9)}$$

$$d_{BL} = a\sqrt{3u^2 - \frac{11}{4}u + \frac{43}{64}} \cdots\cdots\cdots \textbf{(A10)}$$

$$d_{AE} = a\sqrt{2}(2u - 0.5) \cdots\cdots\cdots \textbf{(A11)}$$

$$d_{BE} = a\sqrt{2}(1 - 2u) \cdots\cdots\cdots \textbf{(A12)}$$

$$d_{BAU} = a\sqrt{4u^2 - 3u + \frac{11}{16}} \cdots\cdots\cdots \textbf{(A13)}$$

Equations for various bond length and bond angles [1]:

Me-Me distances

$$b = \frac{a\sqrt{2}}{4} \cdots\cdots\cdots \textbf{(A14)}$$

$$c = \frac{a\sqrt{11}}{8} \cdots\cdots\cdots \textbf{(A15)}$$

$$d = \frac{a\sqrt{3}}{4} \cdots\cdots\cdots \textbf{(A16)}$$

$$e = \frac{3\sqrt{3}a}{8} \cdots\cdots\cdots \textbf{(A17)}$$

$$f = \frac{a\sqrt{6}}{4} \cdots\cdots\cdots \textbf{(A18)}$$

Me-O distances

$$p = a\left(\frac{5}{8} - u\right) \cdots\cdots\cdots \quad \textbf{(A19)}$$

$$q = a\sqrt{3}\left(u - \frac{1}{4}\right) \cdots\cdots\cdots \quad \textbf{(A20)}$$

$$r = \frac{a\sqrt{11}u}{4} \cdots\cdots\cdots \quad \textbf{(A21)}$$

$$s = a\sqrt{3}\left(\frac{u}{3} + \frac{1}{8}\right) \cdots\cdots\cdots \quad \textbf{(A22)}$$

Bond angles

$$\theta_1 = \cos^{-1}\left(\frac{p^2 + q^2 - c^2}{2pq}\right) \cdots\cdots\cdots \quad \textbf{(A23)}$$

$$\theta_2 = \cos^{-1}\left(\frac{p^2 + r^2 - e^2}{2pr}\right) \cdots\cdots\cdots \quad \textbf{(A24)}$$

$$\theta_3 = \cos^{-1}\left(\frac{p^2 - b^2}{2p^2}\right) \cdots\cdots\cdots \quad \textbf{(A25)}$$

$$\theta_4 = \cos^{-1}\left(\frac{p^2 + s^2 - f^2}{2ps}\right) \cdots\cdots\cdots \quad \textbf{(A26)}$$

$$\theta_5 = \cos^{-1}\left(\frac{r^2 + q^2 - d^2}{2qr}\right) \cdots\cdots\cdots \quad \textbf{(A27)}$$

Equations for the ionic packing coefficient P_{tet} and P_{oct} at the tetrahedral and octahedral sites [34]:

$$P_{tet} = \frac{R_{tet} - R_o}{r_{tet}} \cdots\cdots\cdots \quad \textbf{(A28)}$$

$$P_{oct} = \frac{R_{oct} - R_o}{r_{oct}} \cdots\cdots\cdots \quad \textbf{(A29)}$$

Here, $R_{tet}(= d_{AL})$ and $R_{tet}(= d_{BL})$ are the average bond length at tetrahedral and octahedral sites respectively.

Equation for the fulfillment coefficient (α) of the unit cell using the following relation [34]**:**

$$\alpha = \frac{32\pi\left(r_{tet}^3 + 2r_{oct}^3 + 4R_o^3\right)}{3a_{exp}^3} \cdots\cdots\cdots \quad \textbf{(A30)}$$

Equation of vacancy parameter β [34]:

$$\beta = \frac{a_{th}^3 - a_{exp}^3}{a_{th}^3} \times 100\% \cdots\cdots\cdots \quad \textbf{(A31)}$$

Equation for tolerance factor calculation [36]:

$$T = \frac{1}{\sqrt{3}}\left[\frac{r_{tet} + R_O}{r_{oct} + R_O}\right] + \frac{1}{\sqrt{2}}\left[\frac{R_O}{r_{tet} + R_O}\right] \cdots\cdots\cdots \quad \textbf{(A32)}$$

The FWHM data has been calculated by using the following relation [37]:

$$\beta_O = 0.5346 \times W_L + \sqrt{0.2166 W_L^2 + W_G^2} \cdots\cdots\cdots \quad \textbf{(A33)}$$

Here, β_O is observed FWHM, W_L, and W_G is the Lorentzian and Gaussian width respectively. The instrumental broadening (β_i) removal equation [34]:

$$\beta_{hkl} = [(\beta_O - \beta_i)(\beta_O^2 - \beta_O^2)^{0.5}]^{0.5} \cdots\cdots\cdots \quad \textbf{(A34)}$$

The Scherrer equation [17]:

$$D = \frac{0.9\lambda}{\beta_{311}\cos\theta} \cdots\cdots\cdots \quad \textbf{(A35)}$$

Here, β_{311} is the FWHM at (311) plane (most intense peak), λ (=1.5406 Å) represents the wavelength of incident X-rays.

Modified Scherrer equation [22]:

$$ln\beta = ln\left(\frac{0.9\lambda}{D}\right) + ln\left(\frac{1}{\cos\theta}\right) \cdots\cdots\cdots \quad \textbf{(A36)}$$

Williamson–Hall relation [14, 21]:

$$\beta_{tot} = \beta_{strain} + \beta_{crystal} = 4\varepsilon tan\theta + \frac{0.9\lambda}{Dcos\theta}$$

Or,

$$\beta_{hkl} cos\theta = 4\varepsilon sin\theta + \frac{0.9\lambda}{D} \cdots\cdots\cdots \quad \textbf{(A37)}$$

Here, β_{hkl} is the total broadening due to strain and size in a particular peak having the (hkl) value which is written exchange of β_{tot}.

Equation for the size strain plot [21]:

$$\left(d_{hkl}.\beta_{hkl}.cos\theta\right)^2 = \frac{K\lambda}{D}\left(d_{hkl}^2\beta_{hkl}.cos\theta\right) + \frac{\varepsilon^2}{4}\cdots\cdots\cdots \quad \textbf{(A38)}$$

Here, d_{hkl} is lattice spacing for different (hkl) planes.

Force constant according to Waldron relation [21]:

$$k_t = 4\pi^2c^2\nu_1^2\mu\cdots\cdots\cdots \quad \textbf{(A39)}$$

$$k_o = 4\pi^2c^2\nu_2^2\mu\cdots\cdots\cdots \quad \textbf{(A40)}$$

$$k_{av} = (k_t + k_o)/2\cdots\cdots\cdots \quad \textbf{(A41)}$$

Here, c is the velocity of light, μ is the reduced mass which we have calculated by the following relation [38, 39]:

$$\mu = \frac{m_1 * m_2}{m_1 + m_2}\cdots\cdots\cdots \quad \textbf{(A42)}$$

Where m_1 is the weighted average of atomic weights of the cations residing at the A-site or B-site while m_2 is the atomic weight of oxygen anion.

Equation for the stiffness constant C_{11}, C_{12} [21]:

$$C_{11} = \frac{k_{av}}{a}\cdots\cdots\cdots \quad \textbf{(A43)}$$

$$C_{12} = \frac{\sigma C_{11}}{1-\sigma}\cdots\cdots\cdots \quad \textbf{(A44)}$$

Here, σ is the Poisson's ratio as a function of pore fraction P. The corresponding relation for σ is as follows [21]:

$\sigma = 0.324(1 - 1.043P)\cdots\cdots\cdots (A45)$

Some elastic properties related equations [40, 41]:

Bulk modulus:

$$B = \frac{1}{3}(C_{11} + 2C_{12}) \cdots\cdots\cdots \quad \textbf{(A46)}$$

Longitudinal wave velocity,

$$v_l = \sqrt{\frac{C_{11}}{d_x}}, \cdots\cdots\cdots \quad \textbf{(A47)}$$

Here, d_x is the X-ray density

Transverse wave velocity,

$$v_t = \frac{v_l}{\sqrt{3}} \cdots\cdots\cdots \quad \textbf{(A48)}$$

Rigidity modulus,

$$G = d_x v_t^2 \cdots\cdots\cdots \quad \textbf{(A49)}$$

Young's modulus,

$$E = 2(1 + \sigma)G \cdots\cdots\cdots \quad \textbf{(A50)}$$

Mean velocity,

$$v_m = \left(\frac{1}{3}\left(\frac{2}{v_t^3} + \frac{1}{v_l^3}\right)\right)^{-1/3} \cdots\cdots\cdots \quad \textbf{(A51)}$$

Hasselman and Fularth's formulae for the corrected zero porosity elastic moduli [41, 21]:

$$\frac{1}{E_o} = \frac{1}{E}\left[1 - \frac{3p(1-\sigma)(9+5\sigma)}{2(7-5\sigma)}\right] \cdots\cdots\cdots \quad \textbf{(A52)}$$

$$\frac{1}{G_o} = \frac{1}{G}\left[1 - \frac{15p(1-\sigma)}{7-5\sigma}\right] \cdots\cdots\cdots \quad \textbf{(A53)}$$

$$B_o = \left[\frac{E_o G_o}{3(3G_o - E_o)}\right] \cdots\cdots\cdots \quad \textbf{(A54)}$$

$$\sigma_o = \left(\frac{E_o}{2G_o}\right) - 1 \cdots\cdots\cdots \quad \textbf{(A55)}$$

Anderson's formula for the Debye temperature, θ_D [44]:

$$\theta_D = \frac{h}{k_B}\left(\frac{3qd_x N_A}{4\pi M}\right)^{\frac{1}{3}} v_m \cdots\cdots\cdots \quad \textbf{(A56)}$$

Where h is Planck's constant, k_B is Boltzmann's constant, N_A is Avogadro's number, d_x is the density, M is the molecular mass, and q is the number of atoms per unit formula.

Equations for thermal conductivity [38]:

$$K_{min} = k_B v_m \left(\frac{M}{q d_x N_A}\right)^{-2/3} \text{.........} \quad \textbf{(A57)}$$

Appendix B

Table-B1. Possible cation distribution for all samples of series S1($Co_{1-x}Cr_xFe_2O_4$) and S2 ($Co_{1+x}Cr_xFe_{2-x}O_4$).

Series →		S1					S2			
**x →**		**0.000**	**0.125**	**0.250**	**0.375**	**0.500**	**0.125**	**0.250**	**0.375**	**0.500**
**Tetrahedral Site(± 2%)**	**Co**	0.132	0.100	0.090	0.070	0.050	0.179	0.121	0.042	0.012
	**Cr**	0	0.057	0.110	0.166	0.232	0.011	0.012	0.018	0.008
	**Fe**	0.868	0.843	0.800	0.764	0.718	0.810	0.867	0.940	0.980
**Octahedral Site (± 2%)**	**Co**	0.868	0.775	0.660	0.555	0.450	0.946	1.129	1.333	1.488
	**Cr**	0	0.068	0.140	0.209	0.268	0.114	0.238	0.357	0.492
	**Fe**	1.132	1.157	1.200	1.236	1.282	1.065	0.883	0.685	0.520
**Calculated**	**I_{400}/I_{440}**	0.77	0.76	0.81	0.81	0.83	0.83	0.85	0.80	0.80
	**I_{220}/I_{440}**	0.98	0.82	1.11	1.05	1.06	1.14	1.08	1.05	0.80
**Observed**	**I_{400}/I_{440}**	0.74	0.75	0.72	0.76	0.78	0.84	0.90	0.78	0.77
	**I_{220}/I_{440}**	1.03	1.03	1.10	1.02	1.01	1.25	1.24	1.00	1.04
**Inversion parameter (y)**		0.76	0.72	0.66	0.62	0.56	0.76	0.98	1.37	1.88
**Ionic Radius (Å)**	**r_{tet}**	0.63	0.64	0.65	0.66	0.68	0.64	0.63	0.62	0.61
	**r_{oct}**	0.69	0.68	0.68	0.67	0.66	0.73	0.78	0.83	0.87

Table-B2. Lattice parameter, density, and porosity for all samples of series S1($Co_{1-x}Cr_xFe_2O_4$) and S2 ($Co_{1+x}Cr_xFe_{2-x}O_4$).

Series	x	a_{exp} (Å)	V_{exp} (Å^3)	a_{true} (Å)	V_{true} (Å^3)	a_{th} (Å)	V_{th} (Å^3)	d_x (g/cm^3)	d_B (g/cm^3)	d_{reit} (g/cm^3)	P(%)
S1	0	8.3492	582.01	8.3539	582.45	8.3635	585	5.36	4.90	5.34	9
	0.125	8.3520	582.6	8.3596	579.51	8.3642	585	5.33	4.87	5.33	9
	0.250	8.3542	583.06	8.3600	578.15	8.3647	585	5.31	4.78	5.29	10
	0.375	8.3644	585.2	8.3672	577.47	8.3658	586	5.27	4.69	5.27	11
	0.500	8.3765	587.74	8.3746	576.55	8.3713	587	5.31	4.61	5.22	13
S2	0.125	8.3556	582.39	8.3705	586.48	8.4927	613	5.52	4.94	5.52	11
	0.250	8.3581	589.32	8.3896	590.50	8.6096	638	5.67	5.00	5.68	12
	0.375	8.3649	595.80	8.3958	591.81	8.7272	665	5.82	5.07	5.85	13
	0.500	8.376	604.68	8.4061	593.99	8.8414	691	5.95	5.09	6.01	15

Table-B3. Values of hopping lengths (L_A, L_B), Tetrahedral bond length (d_{AL}), octahedral bond lengths (d_{BL}) , tetrahedral edge length (d_{AE}), shared and unshared octahedral edge (d_{BE}, d_{BEU}) , interatomic bond lengths and angles for all samples of series S1($Co_{1-x}Cr_xFe_2O_4$) and S2 ($Co_{1+x}Cr_xFe_{2-x}O_4$).

Series →		S1					S2			
**x →**		**0.000**	**0.125**	**0.250**	**0.375**	**0.500**	**0.125**	**0.250**	**0.375**	**0.500**
**L_A (Å)**		3.623	3.622	3.617	3.616	3.614	3.667	3.717	3.768	3.809
**L_B (Å)**		2.958	2.957	2.953	2.952	2.951	2.994	3.035	3.077	3.11
**d_{AL} (Å)**		1.945	1.956	1.968	1.982	1.998	1.926	1.893	1.86	1.832
**d_{BL} (Å)**		2.011	2.006	2.000	1.996	1.993	2.023	2.042	2.063	2.083
**d_{AE} (Å)**		3.176	3.194	3.215	3.237	3.263	3.145	3.091	3.037	2.991
**d_{BE} (Å)**		2.727	2.711	2.692	2.677	2.659	2.762	2.818	2.877	2.931
**d_{BEU} (Å)**		2.956	2.958	2.959	2.964	2.969	2.957	2.956	2.958	2.961
**Me-Me distances (Å)**	**b**	2.9569	2.9572	2.9574	2.9577	2.9597	3.0026	3.0439	3.0855	3.1259
	**c**	3.4673	3.4676	3.4678	3.4683	3.4705	3.5209	3.5693	3.6181	3.6654
	**d**	3.6215	3.6218	3.6220	3.6225	3.6248	3.6774	3.7280	3.7789	3.8284
	**e**	5.4322	5.4327	5.4330	5.4337	5.4373	5.5162	5.5921	5.6684	5.7426
	**f**	5.1216	5.1220	5.1223	5.1230	5.1263	5.2007	5.2723	5.3442	5.4142
**Me-O distances (Å)**	**p**	2.0113	2.0056	1.9987	1.9923	1.9861	2.0543	2.1027	2.1523	2.1990
	**q**	1.9484	1.9588	1.9712	1.9829	1.9973	1.9579	1.9500	1.9405	1.9337
	**r**	3.7310	3.7508	3.7745	3.7970	3.8245	3.7491	3.7340	3.7158	3.7028
	**s**	3.6674	3.6711	3.6754	3.6797	3.6865	3.7171	3.7567	3.7959	3.8349
**Bond Angles (degree)**	θ_1	122.23	122.00	121.74	121.49	121.21	122.67	123.41	124.17	124.85
	θ_2	140.27	139.39	138.35	137.40	136.35	142.08	145.27	148.86	152.38
	θ_3	94.62	94.98	95.43	95.85	96.33	93.90	92.73	91.58	90.58

	θ_4	126.30	126.38	126.47	126.56	126.66	126.14	125.89	125.63	125.40
	θ_5	71.54	70.98	70.31	69.68	68.99	72.70	74.68	76.80	78.75

Table-B4. Oxygen positional parameter, ionic packing coefficient, fulfillment coefficient, vacancy parameter, and tolerance factor for all samples of series S1($Co_{1-x}Cr_xFe_2O_4$) and S2 ($Co_{1+x}Cr_xFe_{2-x}O_4$).

Series	x	Oxygen Positional Parameter, u and Deviation, δ		Ionic Packing Coefficient		Fulfillment Coefficient α	Vacancy parameter β	Tolerance factor T
		u	δ	P_a	P_b			
S1	0	0.3845	0.0095	0.9867	0.9967	0.5819	0.5145	1.038
	0.125	0.3852	0.0102	0.9877	0.9982	0.5811	0.4370	1.040
	0.250	0.3860	0.0110	0.9884	0.9988	0.5804	0.3783	1.043
	0.375	0.3868	0.0118	0.9919	0.9991	0.5782	0.0526	1.045
	0.500	0.3877	0.0127	0.9944	1.0008	0.5758	-0.1860	1.048
S2	0.125	0.3831	0.0081	0.9426	0.9589	0.5887	4.7671	1.027
	0.250	0.3807	0.0057	0.9016	0.9239	0.5972	8.5123	1.014
	0.375	0.3784	0.0033	0.8621	0.8940	0.6062	11.9433	1.000
	0.500	0.3763	0.0013	0.8259	0.8692	0.6150	14.9758	0.990

Table-B5. Crysttalite size (D) and strain (ε) estimated by different methods for all samples of series S1($Co_{1-x}Cr_xFe_2O_4$) and S2 ($Co_{1+x}Cr_xFe_{2-x}O_4$).

**Series**	**x**	**Scherrer Method**	**Modified Scherrer Method**	**Rietveld refined Method**		**Williamson–Hall Method**		**Size Strain Method**	
		**D (nm)**	**D (nm)**	**D (nm)**	ε	**D (nm)**	ε	**D (nm)**	ε
**S1**	0	46	53	61	0.00092	71	0.000725	60	1.41×10^{-05}
	0.125	46	51	57	0.00085	70	0.000746	52	5.91×10^{-06}
	0.250	45	49	52	0.00071	64	0.000775	51	9.3510^{-06}
	0.375	39	45	51	0.00058	60	0.000909	50	1.33×10^{-05}
	0.500	39	43	46	0.00046	56	0.000905	47	1.40×10^{-05}
**S2**	0.125	47	50	59	0.00075	67	0.000786	58	1.14×10^{-05}
	0.250	40	43	49	0.00068	52	0.000624	45	5.94×10^{-05}
	0.375	37	40	41	0.00051	43	0.000374	42	5.55×10^{-06}
	0.500	39	39	38	0.00046	43	0.000353	40	2.21×10^{-06}

Table-B6. Absorbtion wave number (ν_1, ν_2), force constant, stiffness constant, wave velocity, elastic constant, Pugh ratio, Poisson ratio Debye temperature, and minimum thermal constant for all samples of series S1($Co_{1-x}Cr_xFe_2O_4$) and S2 ($Co_{1+x}Cr_xFe_{2-x}O_4$).

Series ⟶		S1					S2			
x ⟶		0	0.125	0.250	0.375	0.500	0.125	0.250	0.375	0.500
ν_1 (cm^{-1})		371	373	375	377	378	374	376	377	378
ν_2(cm^{-1})		554	558	564	568	572	586	590	594	598
**Force Constant**	k_t (N/m)	290	294	300	305	309	324	329	333	338
	k_o(N/m)	130	131	133	134	135	132	133	134	135
	k_{ab}(N/m)	210	213	217	219	222	228	231	234	236
**Stiffness Costant**	C_{11}(GPa)	251	255	260	264	268	273	277	280	284
	C_{12}(GPa)	105	106	106	106	106	110	110	109	108
**Wave Velocity**	v_l (ms^{-2})	6843	6911	6999	7080	7158	7035	6986	6940	6912
	v_t(ms^{-2})	3950	3990	4041	4087	4133	4062	4033	4007	3991
	v_m(ms^{-2})	4156	4197	4250	4299	4348	4272	4243	4215	4198
**Elastic Constant**	B(GPa)	153	156	157	158	160	164	165	166	167
	G (Gpa)	84	84	87	88	89	91	92	93	95
	E (GPa)	216	220	223	226	229	234	237	239	241
**Pugh ratio (B/G)**		1.84	1.83	1.82	1.80	1.79	1.81	1.79	1.78	1.76
σ		0.29	0.29	0.29	0.29	0.28	0.29	0.28	0.28	0.28
**Zero Porosity Elastic Constant**	Eo (GPa)	261	266	280	291	300	298	310	322	340
	Go (Gpa)	100	102	107	111	115	114	119	124	131
	Bo (GPa)	226	230	238	244	250	252	259	265	275
σ_0		0.31	0.31	0.30	0.30	0.30	0.30	0.30	0.29	0.29
**Bo/Go**		2.26	2.25	2.22	2.19	2.16	2.20	2.16	2.14	2.09
θ_D (K)		593	599	606	612	618	610	605	601	598
K_{min}		1.31	1.33	1.34	1.36	1.37	1.35	1.34	1.33	1.32
